# A clinical and experimental investigation of liraglutide effects on the brain-kidney axis

**DOI:** 10.1126/sciadv.aef4951

**Published:** 2026-08-05

**Authors:** Michael P. Greenwood, Mohammed I. Alotaibi, Soledad Bárez-López, Nathan W. P. Cantley, Sharon Merritt, Danijela Tatovic, David Murphy

**Affiliations:** ^1^ Molecular Neuroendocrinology Research Group, Bristol Medical School: Translational Health Sciences, University of Bristol, Dorothy Hodgkin Building, Bristol, UK.; ^2^ School of Physiology, Pharmacology and Neuroscience, University of Bristol, Biomedical Sciences Building, Bristol, UK.; ^3^ Department of Medical Biochemistry, College of Medicine, Taif University, Taif 21944, Saudi Arabia.; ^4^ Instituto de Investigaciones Biomédicas Sols-Morreale, Consejo Superior de Investigaciones Científicas (CSIC) - Universidad Autónoma de Madrid (UAM), Madrid, Spain.; ^5^ Department of Clinical Biochemistry, North Bristol NHS Trust, Bristol, UK.; ^6^ Diabetes Research Unit, North Bristol NHS Trust, Bristol, UK.; ^7^ Diabetes and Endocrinology Department, North Bristol NHS Trust, Bristol, UK.; ^8^ Department of Immunology and Immunotherapy, College of Medicine and Health, University of Birmingham, Birmingham, UK.

## Abstract

Glucagon-like peptide-1 receptor agonists elicit diuresis and natriuresis in humans and rodents. However, little is known about their interactions with the bodies hormonal systems that control fluid homeostasis. We performed a single center, open-label, before-and-after study and discovered that liraglutide reduces plasma arginine vasopressin (AVP) levels in healthy humans. By quantitative proteomic and phosphoproteomic investigation of the rat pituitary gland, we detail time- and sex-dependent modifications to synaptic proteins mediated by liraglutide. We developed an in vitro AVP luciferase assay to assess the impact of synaptic protein phosphorylation on AVP secretion. We used this assay to identify synaptic protein phosphosites that influence AVP release and liraglutide-mediated sex differences in AVP release. Investigation of AVP downstream signaling pathways in the kidney revealed posttranslational changes to the crucial AVP-regulated water channel aquaporin 2. Thus, glucagon-like peptide-1 receptor agonist modulation of AVP release may be responsible for cardiovascular and renal changes observed in patients.

## Teaser

Liraglutide affects renal function by altering vasopressin secretion.

## INTRODUCTION

As water is so essential to life, it is necessary to understand drug actions on the major axes controlling body fluid homeostasis in health and disease. The incretin hormone glucagon-like peptide-1 (GLP-1) produced naturally by intestinal L cells and in the brain is involved in glucose homeostasis via stimulation of insulin secretion as well as acting as a satiety factor (*1*). The short half-life of endogenously produced GLP-1 has led to the formulation of stable longer-acting agonists (GLP-1RA) targeting its receptor, GLP-1R (*2*–*4*). In the clinic, such agonists are now widely used to address the major public health crises of diabetes and obesity. However, we still know very little about the interactions of drugs targeting the GLP-1R with the systems that control fluid homeostasis.

The major mechanism of water homeostasis in animals and humans is the control of urinary free water excretion, which is dictated by the circulating plasma levels of the antidiuretic hormone arginine vasopressin (AVP). In the hypothalamus, magnocellular neurons located in the paraventricular nucleus (PVN) and supraoptic nucleus (SON) make AVP that is transported along axons to the posterior pituitary (PP) where it is stored in neurosecretory granules in nerve terminals until specific stimuli stimulate secretion into the peripheral circulation (*5*). Antidiuresis occurs via interaction with the vasopressin 2 receptors in the kidney, and this results in increased water uptake that is mediated by the translocation of aquaporin 2 (AQP2) water channels to the apical membrane of collecting duct principal cells (*6*). This involves AVP-modulated changes to *Aqp2* gene transcription and posttranslational changes by phosphorylation of the AQP2 protein (*7*). In the distal nephron, AVP activation of the epithelial Na^+^ channel promotes sodium reabsorption that creates an osmotic gradient for maximal water reabsorption (*8*). Thus, AVP decreases water excretion and, by promoting sodium reabsorption, decreases sodium excretion. The importance of AVP to water homeostasis is highlighted by the pathophysiology that ensues when AVP or its receptor targets become altered in their abundance, for example, congenital nephrogenic diabetes insipidus (*9*, *10*).

Changes to water homeostasis can be propagated via several well reported mechanisms of GLP-1RA. These agonists decrease both thirst sensation and appetite in rodents and humans, the outcome being that less fluid enters the body (*11*–*13*). They slow gastric emptying, and absorption of water and solutes occurs predominantly in the small intestine so the outcome will be an effect on the rate of water absorption following ingestion (*14*). They increase sodium excretion by the kidney, and one widely reported mechanism involves decreased activity of Na(+)/H(+) exchanger, NHE3 (*15*, *16*). The resulting decrease in proximal tubular reabsorption of sodium promotes diuresis and natriuresis with an outcome of increased water loss. In addition, common side effects of GLP-1RA include nausea, vomiting, and diarrhea, which can lead to dehydration due to increased water loss (*17*). In some case reports, GLP-1RA liraglutide has been associated with acute kidney injury, and the pathological mechanisms have been based on acute tubular necrosis because of dehydration (*18*, *19*). Manufacturers and doctors already stipulate the need to maintain fluid intake with GLP-1RA. In these ways, GLP-1RA plays a notable role in disturbing water homeostasis, but little is known about their interactions with the bodies major hormonal control mechanism, AVP.

We have shown that GLP-1RA liraglutide inhibits the synthesis and secretion of AVP in the rat and shown that this response could be mediated directly by GLP-1Rs located in AVP neuron nerve terminals in the PP (*20*, *21*). However, the actions of GLP-1RA on AVP secretion remain controversial in humans and rodents. Zueco and colleagues reported reduced secretion in awake rats, whereas Bojanowska and Stempniak reported increased secretion (*22*, *23*). The later study was conducted on anesthetized rats, which has been shown to alter neuronal activity and signaling pathways in the SON so different approaches may account for this discrepancy (*24*, *25*). In healthy humans, Baretić *et al.* (*26*) reported no change in plasma AVP during an acute GLP-1 infusion protocol. Thus, the first aim of the present study was to address this conflict in our knowledge of GLP-1RA interactions with AVP secretion in humans.

We performed a single center, open-label, before-and-after study in humans with liraglutide to explore the translational possibility of GLP-1RA liraglutide altering AVP secretion. We assessed the effects of a longer duration of liraglutide treatment on the rat AVP system, which is of clinical importance as GLP-1RA are used continuously to treat chronic diseases of diabetes and obesity. Due to the sheer abundance of GLP-1R in PP nerve terminals (*20*, *23*), and their largely unexplored actions on hormone release by this crucial axis, we performed quantitative proteomics and phosphoproteomics of the male and female pituitary, comparing short and longer-term liraglutide treatments. The data led us to develop tools and assays to study how synaptic protein phosphorylation affects hormone storage and release.

## RESULTS

### Liraglutide treatment decreases plasma AVP and copeptin levels in healthy human subjects

**Fig. 1.**
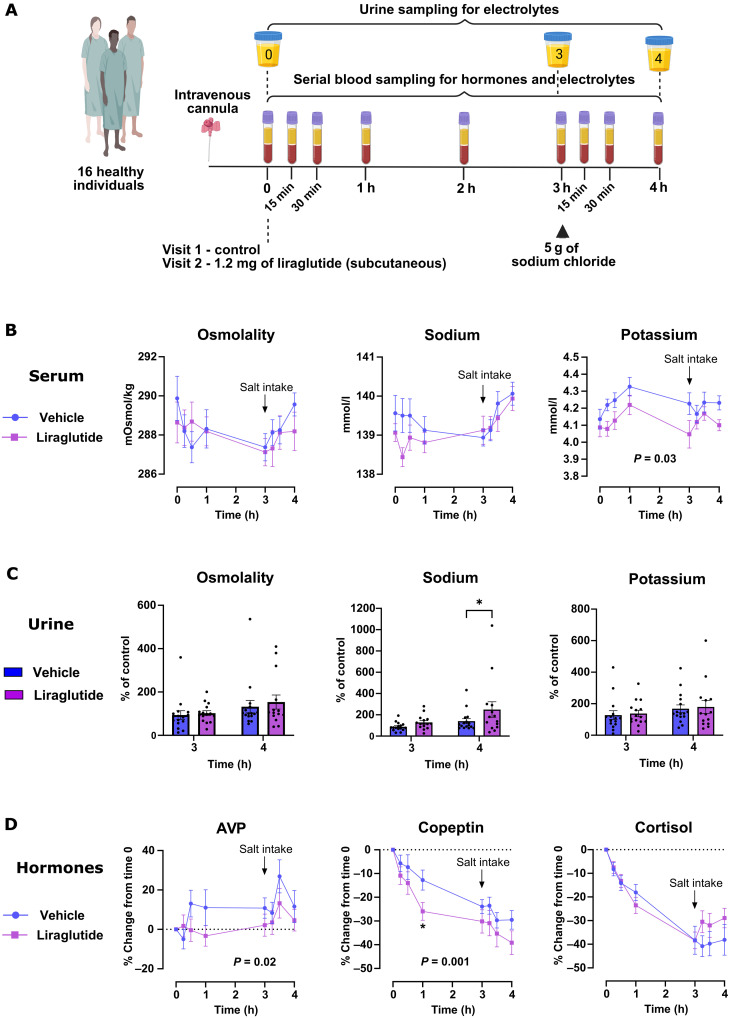
GLP-1RA liraglutide rapidly reduces circulating AVP and copeptin levels in healthy human subjects. (**A**) Outline of the experimental protocol for the human clinical trial. (**B**) Measures of serum osmolality, sodium, and potassium. (**C**) Urine osmolality, sodium, and potassium calculated as percentage of baseline values (time 0 = 100%) for each participant in the trial. (**D**) Plasma levels of AVP, copeptin, and cortisol as determined by enzyme-linked immunosorbent assay (ELISA) and presented as percentage change from baseline (time 0) for each participant of the trial. The timing of the oral salt load is indicated by a black downward facing arrow in (B) and (D). Statistical analyses were performed by mixed-effects analysis (B) and two-way analysis of variance (ANOVA) (C and D) with Fisher’s post hoc analyses. Values are means ± SEM of *n* = 16 trial participants. ^∗^*P* ≤ 0.05. (A) Created in BioRender. M. Greenwood (2026), https://BioRender.com/9i9ngy8. h, hours.

A protocol diagram for our human study is shown in Fig. 1A. Due to blocked cannula, the series of sample collection for two participants (one female and one male) could not be completed for their second study visit. Therefore, only 16 of the participants recruited completed the trial and were taken forward for further analysis. The median age of participants was 34.5 with a median body mass index of 25.3 (table S1). Baseline values for blood and urine measures can be found in table S2. We looked for any effects of liraglutide on serum, urine, and hormone measures. There were no significant effects of treatment on plasma osmolality or sodium, but there was a significant effect on plasma potassium with levels appearing lower after treatment (Fig. 1B). Notably, a recent study reported that the use of GLP-1RA was associated with lower hyperkalemia risk in patients with chronic kidney disease, and some clinical studies have suggested mechanisms that promote potassium excretion (*27*). There was no significant effect of treatment on urine osmolality or potassium, but there was a significant effect on urine sodium levels (Fig. 1C). Increased urinary sodium concentration is consistent with reported natriuretic functions of GLP-1RA (*16*). There was a significant effect of treatment on AVP and copeptin, but not cortisol levels (Fig. 1D). Copeptin and cortisol levels in humans are known to decline throughout the morning in agreement with our data (*28*). Unlike AVP, the oral salt load did not increase copeptin levels, which may be due to its superior stability, reducing the sensitivity of this surrogate to acute perturbations in release (*29*).

### Liraglutide disturbances to food and water intake in male and female rats

**Fig. 2.**
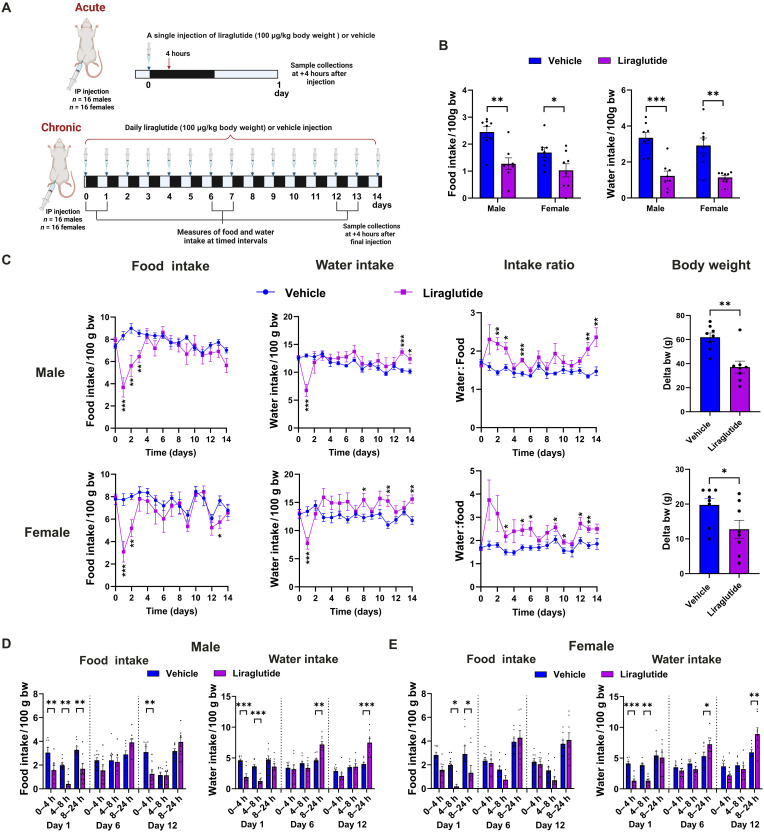
Dynamic fluctuations in food and water intake following GLP-1RA liraglutide treatment in the rat. (**A**) Outline of the experimental protocol. (**B**) Endpoint measures of food intake and water intake in the 4-hour liraglutide treatment groups (acute). (**C**) Daily monitoring of food and water intake for the 14-day liraglutide treatment groups (chronic). The water to food intake ratio is displayed for each day. Differences in body weight gain at the end of study. (**D** and **E**) Measures of food and water intake separated into intervals of 0 to 4, 4 to 8, and 8 to 24 hours on days 1, 6, and 12 of liraglutide treatment. Statistical analyses were performed by independent-sample unpaired *t* tests (B; body weight), repeated measures two-way ANOVA with Fisher’s post hoc (C), and two-way ANOVA with Šídák’s post hoc test (D and E). Values are means ± SEM of *n* = 8 animals per group. ^∗^*P* ≤ 0.05; ^∗∗^*P* ≤ 0.01; ^∗∗∗^*P* ≤ 0.001. (A) Created in BioRender. M. Greenwood (2026), https://BioRender.com/4oy5ri6. bw, body weight; h, hours.

As humans take GLP-1RAs for extended periods, we used the rat to ask about longer-term effects of liraglutide on AVP synthesis and secretion. We investigated the effects after one injection, in line with our earlier inhibitory findings at 4 hours (*20*), and after 14 daily injections as outlined in our experimental protocol (Fig. 2A). As previously observed, liraglutide had significantly reduced food and water intake by 4 hours after administration (Fig. 2B). Physiological data tracking daily food and water intake for 14 days are shown in Fig. 2C. There was a significant effect of treatment on food intake, water intake, and water-to-food intake ratios in males and females. Overall, these data indicated that liraglutide-treated animals may be drinking more water each day than they need.

We analyzed food and water intake across three timed intervals on days 1, 6, and 12 in males (Fig. 2D) and females (Fig. 2E). The significant reduction in food intake was only observed on day 1 of treatment in males and females with one exception being day 12 in males. In contrast, significant effects of treatment on water intake were observed in males and females on days 1, 6 and 12. We show that liraglutide significantly reduced water intake for the first two intervals on day 1 in males and females. In contrast, treatment significantly increased water intake for the third measurement interval on days 6 and 12 in both males and females. These data again indicated disturbances to body fluid with altered patterns of daily fluid intake. Non–body weight corrected data for food and water intake can be found in fig. S1.

### Systematic investigation of distinct components of the AVP system in liraglutide-treated rats

**Fig. 3.**
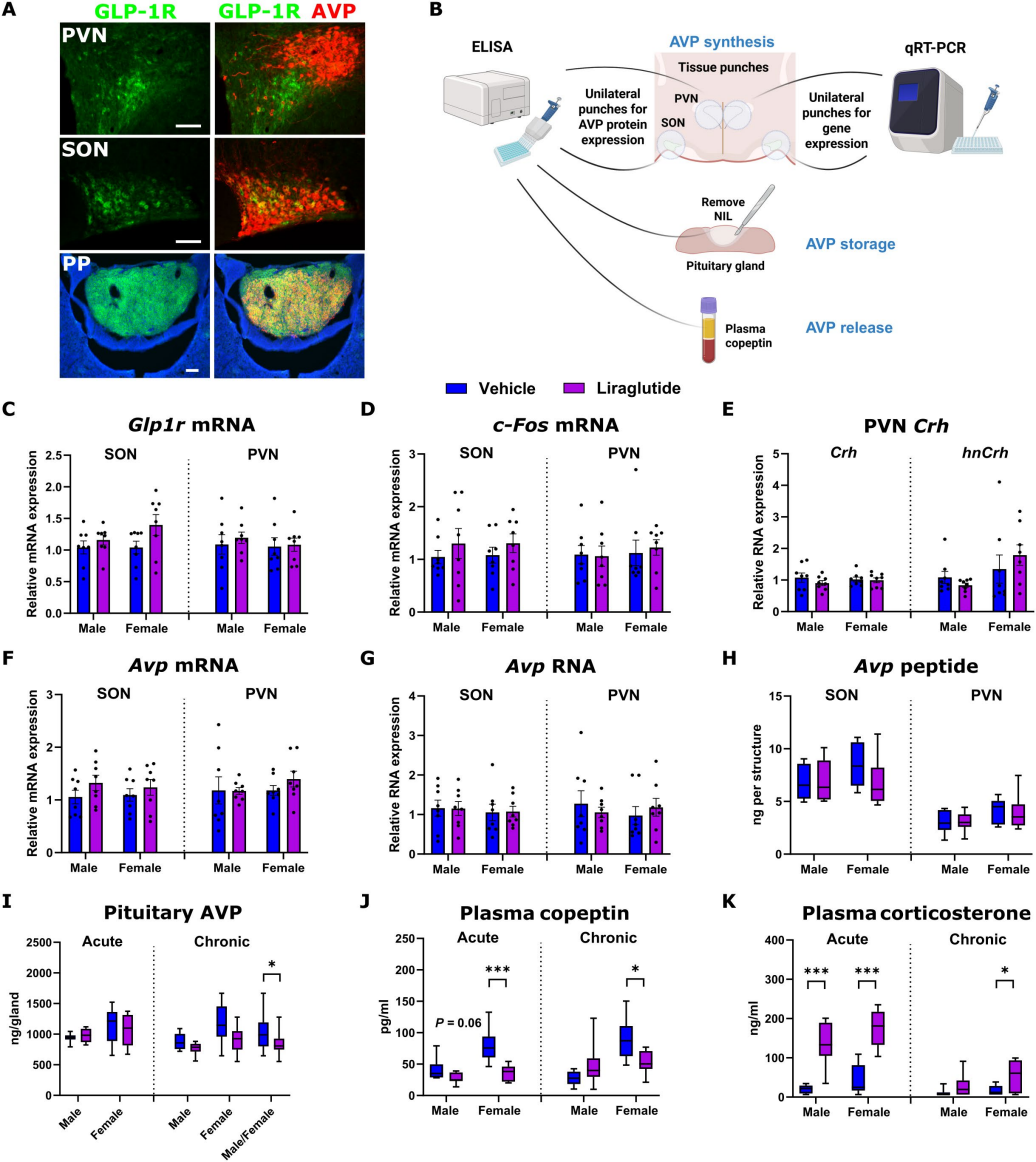
Systematic investigation of distinct components of the AVP system in liraglutide-treated rats. (**A**) Immunostaining for AVP system GLP-1R expression. (**B**) Outline of the experimental system and how measures were collected. Relative RNA expression of *Glp1r* (**C**), *c-Fos* (**D**), *Crh* (**E**), and *Avp* (**F** and **G**) in the SONs and PVNs in control and 14-day liraglutide-treated animals. (**H**) endpoint measures of PVN and SON content of AVP measured by ELISA. (**I**) AVP pituitary stores determined by ELISA. (**J** and **K**) Endpoint measures of plasma copeptin and plasma corticosterone in control and liraglutide-treated animals determined by ELISA. Statistical analyses were performed by independent-sample unpaired *t* tests. Values are means ± SEM of *n* = 7 to 8 per group. ^∗^*P* ≤ 0.05; ^∗∗∗^*P* ≤ 0.001. Acute, 4 hours; chronic, 14 days. Scale bars, 50 μm. (B) Created in BioRender. M. Greenwood (2026), https://BioRender.com/b27fl8x.

We have previously reported several parameters of the acute AVP system response to liraglutide treatment (*20*). Different constituent parts of the AVP system were investigated because of GLP-1R expression throughout this axis (Fig. 3A). Components of the AVP system investigated after 14 days of liraglutide treatment are shown in Fig. 3B. Quantitative reverse transcription polymerase chain reaction (qRT-PCR) showed no change to the expression of *c-Fos*, *Glp1r*, *Crh*, *Avp*, or *hnAvp* in the SON or PVN (Fig. 3, C to G). Notably, we did observe higher *Glp1r* expression in the female PVN, consistent with reports of higher expression in females than that in males (fig. S2) (*30*). We next asked about AVP protein content. In the SON and PVN (Fig. 3H) as well as the PP (Fig. 3I), we found no differences when data were analyzed by sex. However, when PP datasets were combined and analyzed independently of sex, there was a significant decrease in AVP pituitary content (Fig. 3I). There were no significant differences in copeptin in males, although levels were lower at 4 hours, whereas, in females, copeptin was significantly reduced at 4 hours and 14 days (Fig. 3J). We show significantly increased corticosterone levels in male and female rats at 4 hours (Fig. 3K), consistent with the literature (*31*). The magnitude of this response was not replicated at 14 days while remaining significantly elevated in females. To summarize, we show that liraglutide reduces AVP content in the pituitary and reduces circulating levels of copeptin in females.

### Sex and duration of treatment shape the pituitary NIL proteome response to liraglutide

**Fig. 4.**
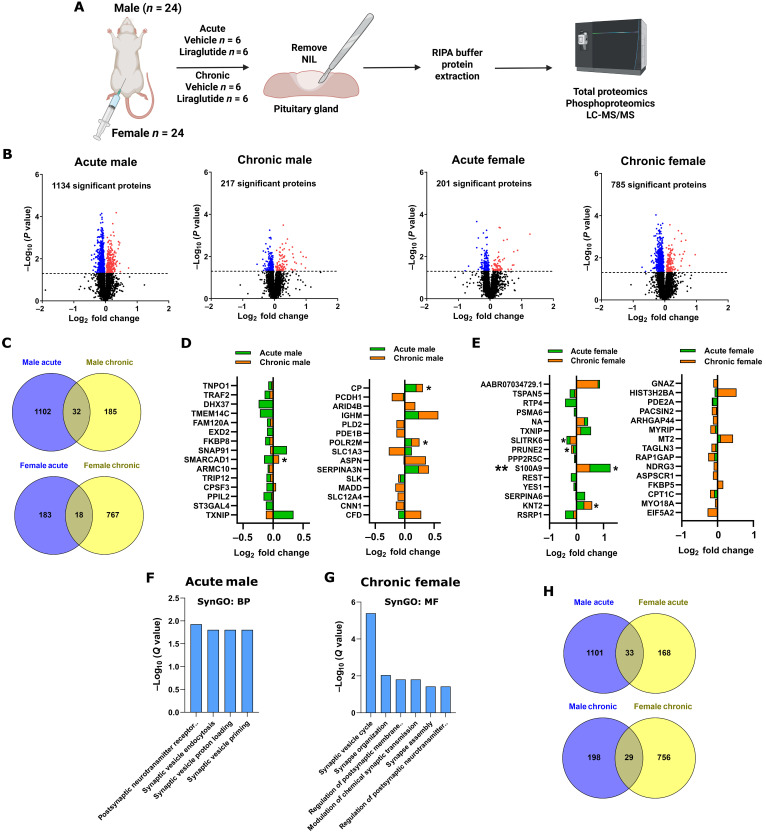
Sex and duration of treatment distinctly shape the pituitary proteome response to liraglutide. (**A**) Outline of the experimental protocol. (**B**) Volcano plots of protein changes in male and female rat NILs in the 4-hour (acute) and 14-day (chronic) liraglutide treatment groups compared to vehicle controls. (**C**) Protein changes for the male and female treatment groups are shown in Venn diagrams. (**D**) Top 15 most significantly altered proteins by *P* value in the male acute and chronic treatment groups each overlaid with data from the other group for comparison. (**E**) Top 15 most significantly altered proteins by *P* value in the female acute and chronic treatment groups each overlaid with data from the other group for comparison. (**F**) Bar chart showing SynGO enriched terms for biological process in acute males displayed as −log_10_ *Q* value. (**G**) Bar chart showing SynGO enriched terms for biological process in chronic females displayed as −log_10_ *Q* value. (**H**) Comparison of protein changes in acute and chronic males and females is shown in overlapping Venn diagrams. For the volcano plots, blue dots indicate decreased protein abundance, and red dots indicate increased protein abundance. *P ≤ 0.05; ** marks the protein used for validation. RIPA, radioimmunoprecipitation assay; BP, biological process; MF, molecular function. (A) Created in BioRender. M. Greenwood (2026), https://BioRender.com/0vupju8.

We asked about the effects of prolonged exposure to liraglutide on the PP, using quantitative proteomics and phosphoproteomics as outlined in our experimental protocol (Fig. 4A). In support of alterations to PP AVP stores, the abundance of AVP prohormone was significantly reduced by liraglutide at day 14 in the female dataset (fig. S3). We show volcano plots to display differentially expressed proteins following liraglutide treatment (Fig. 4B). We identified 1134 significantly altered protein abundances in the neurointermediate lobe (NIL) at 4 hours falling to 217 at 14 days with 32 overlapping proteins in the male (Fig. 4C). In the NIL of the female rat, we identified 201 significantly altered proteins at 4 hours increasing to 785 at 14 days with 18 overlapping proteins (Fig. 4C). We show the top 15 significantly altered proteins in the NIL at 4 hours and 14 days of treatment in males (Fig. 4D) and females (Fig. 4E). To aid dataset comparisons, 4-hour and 14-day data are displayed on the same axis. We next used synaptic Gene Ontologies (SynGO) to mine for synaptic proteins and enriched pathways. We identified four significantly enriched terms for SynGO biological process at 4 hours but no enriched terms for 14 days. The most significantly enriched term was “postsynaptic neurotransmitter receptor activity” (Fig. 4F). In females, there were no significantly enriched terms for SynGO biological process at 4 hours but 24 enriched terms at 14 days. The most significant enriched term being “synaptic vesicle cycle” (Fig. 4G). At 4 hours, there were 33 overlapping proteins and, at 14 days, 29 between males and females (Fig. 4H). We validated the proteomic data by Western blot using S100A9 that increased solely in females (fig. S4). Thus, protein expression changes in the NIL are distinctly different at 4 hours and 14 days and are highly sexually dimorphic.

### Sex and duration of treatment distinctly shape the NIL phosphoproteome response to liraglutide

**Fig. 5.**
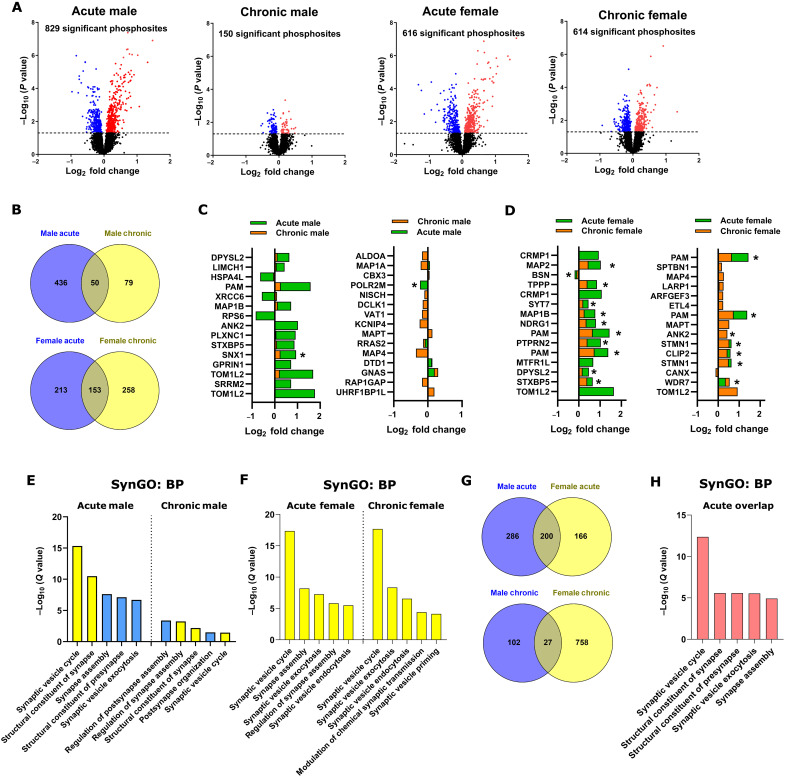
Sex and duration of treatment distinctly shape the pituitary phosphoproteome response to liraglutide. (**A**) Volcano plots of phosphoprotein changes male and female rat NILs in the 4-hour (acute) and 14-day (chronic) liraglutide treatment groups compared to vehicle controls. (**B**) Phosphoprotein changes for the male and female treatment groups are shown in Venn diagrams. (**C**) Top 15 most significantly altered phosphoproteins by *P* value in acute and chronic male groups each overlaid with protein data from the other group for comparison. (**D**) Top 15 most significantly altered phosphoproteins by *P* value in acute and chronic female groups each overlaid with protein data from the other group for comparison. (**E**) Bar chart showing SynGO enriched terms for biological process in acute and chronic males displayed as −log_10_ *Q* value. Yellow bars indicate conserved enriched terms for biological process. (**F**) Bar chart showing SynGO enriched terms for biological process in acute and chronic females displayed as −log_10_ *Q* value. Yellow bars indicate conserved enriched terms for biological process. (**G**) Comparison of phosphoprotein changes in acute and chronic males and females are shown in overlapping Venn diagrams. (**H**) Bar chart showing SynGO enriched terms for biological process for acute male and female overlapping phosphoproteins displayed as −log_10_ *Q* value. For the volcano plots, blue dots indicate decreased protein phosphorylation, and red dots indicate increased protein phosphorylation. BP, biological process.

Posttranslational modification by phosphorylation adds great complexity to the proteome. We investigated how liraglutide affects NIL protein phosphorylation in male and female rats. We show volcano plots to display differentially expressed phosphoproteins in males and females following liraglutide treatment (Fig. 5A). In males, we identified changes to the phosphorylation status of 486 proteins at 4 hours falling to 129 at 14 days with 50 phosphoproteins in common (Fig. 5B). In females, we identified changes to the phosphorylation status of 366 proteins in the NIL at 4 hours and 411 after 14 days, with 153 phosphoproteins in common (Fig. 5B). Of the 15 significant phosphoproteins at 4 hours and 14 days, only one phosphoprotein was found in each comparison (Fig. 5C). Of the 15 most significant phosphoproteins identified at 4 hours, 11 remained significant at 14 days (Fig. 5D). We identified 33 significantly enriched terms for SynGO biological process at 4 hours and 5 at 14 days (Fig. 5E). The most enriched term at 4 hours was “synaptic vesicle cycle” and 14 days “regulation of postsynapse assembly” (Fig. 5E). In females, we identified 34 significantly enriched terms for SynGO biological process at 4 hours and 37 at 14 days. The top Gene Ontology term for 4 hours and 14 days was “synaptic vesicle cycle” (Fig. 5F). Two hundred phosphoproteins were altered in both males and females at 4 hours, suggesting a high level of conservation for this initial posttranslational response to liraglutide (Fig. 5G). By contrast, only 27 phosphoproteins were altered in both males and females at 14 days, highlighting sexual divergence in the response to liraglutide during longer courses of treatment (Fig. 5G). We identified 34 significantly enriched terms for overlapping phosphoproteins between males and females at 4 hours but none after 14 days. Again, the top term for overlapping phosphoproteins was “synaptic vesicle cycle” (Fig. 5H). Thus, we propose that sex differences in protein phosphorylation in the pituitary may explain the lower plasma AVP levels in females.

### Identification of synaptic protein phosphosites that affect AVP release

**Fig. 6.**
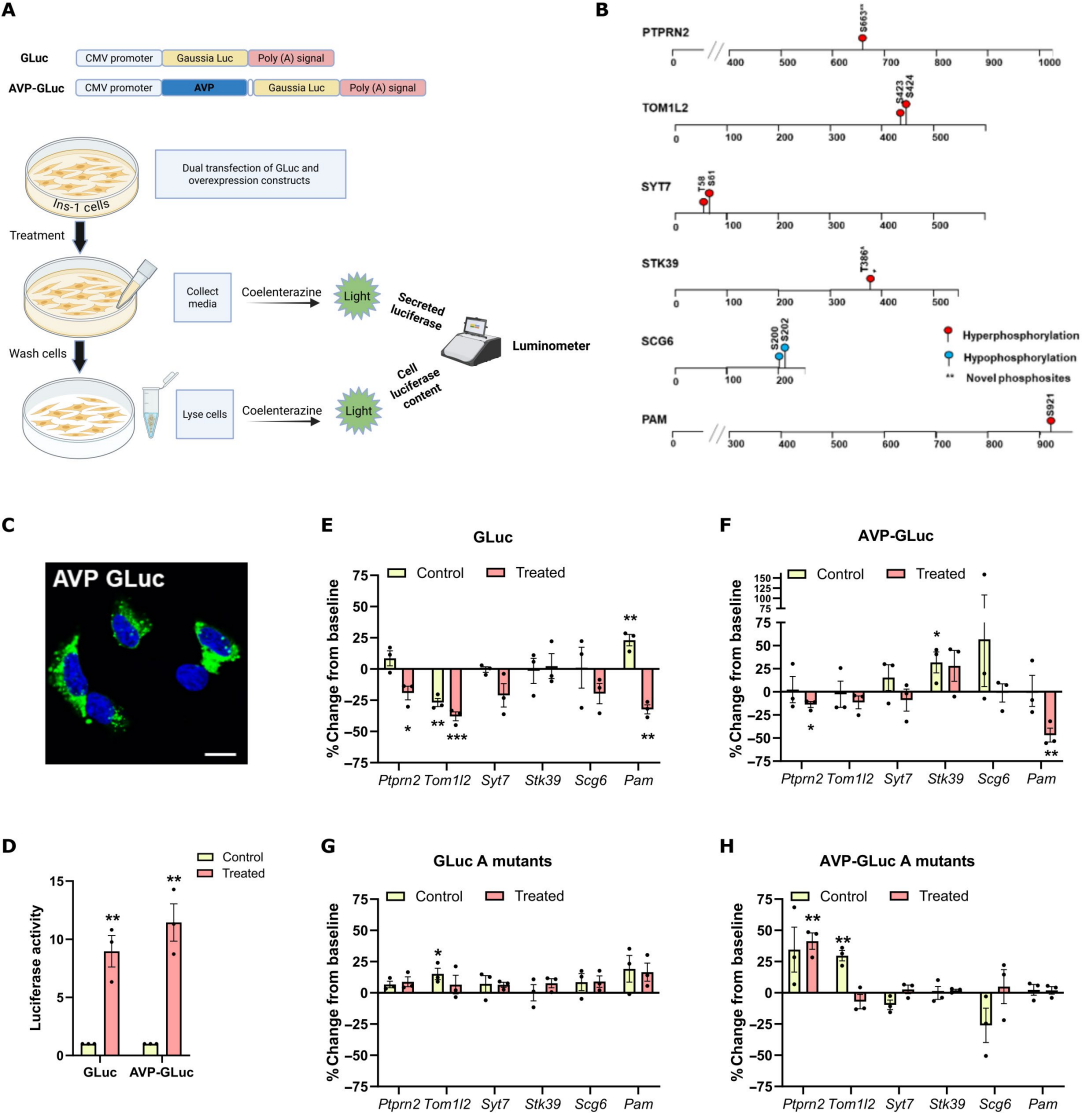
Identification of synaptic protein phosphosites affecting AVP secretion. (**A**) A newly developed Gaussia luciferase (GLuc) system to measure the effects of synaptic protein phosphorylation on AVP secretion. (**B**) Mapping of selected phosphosites undergoing changes to phosphorylation in response to liraglutide treatment compared to vehicle controls. (**C**) Immunofluorescent staining of AVP in Ins-1 cells transfected with AVP-GLuc. (**D**) Gaussia luciferase activity in Ins-1 cell secretions from cells expressing GLuc and AVP-GLuc treated with vehicle or dually with forskolin and PMA. (**E**) Gaussia luciferase activity in Ins-1 cell secretions from cells cotransfected with plasmids expressing synaptic proteins together with GLuc. (**F**) Gaussia luciferase activity in Ins-1 cell secretions from cells cotransfected with plasmids expressing synaptic proteins together with AVP-GLuc. (**G**) Gaussia luciferase activity in Ins-1 cell secretions from cells cotransfected with plasmids expressing phosphonull synaptic proteins together with GLuc. (**H**) Gaussia luciferase activity in Ins-1 cell secretions from cells cotransfected with plasmids expressing phosphonull synaptic proteins together with AVP-GLuc. The secretion from empty pcDNA3.1 transfections was used to set the baseline to 0. The percentage change has then been calculated relative to pcDNA3.1 in control or treatment conditions. Statistical analyses were performed by independent-sample unpaired *t* tests. Values are means ± SEM of *n* = 3 biological replicates per group. *P ≤ 0.05; **P ≤ 0.01; ***P ≤ 0.001. Scale bar, 10 μm. (A) Created in BioRender. M. Greenwood (2026), https://BioRender.com/nkyv1wk.

We created an in vitro secretory system to investigate the impact of specific protein phosphorylation events on AVP secretion (Fig. 6A). The proteins and phosphosites investigated are shown in Fig. 6B. The expression AVP-GLuc localized to the cell cytoplasm in punctate structures, consistent with vesicular expression (Fig. 6C). When stimulated with phorbol 12-myristate 13-acetate (PMA) and forskolin, a robust increase in secretion was observed for AVP-GLuc similar to GLuc alone (Fig. 6D). We selected phosphosites of six different synaptic proteins for investigation—PTPRN2, TOM1L2, SYT7, STK39, GNAS, and PAM—on the basis of the criteria of statistical significance, log_2_ fold change, and sex differences. We first monitored secretion after overexpression of the wild-type clones. The overexpression of *Tom1l2* decreased, whereas *Pam* increased GLuc secretion in the control condition (Fig. 6E). In the stimulated condition, overexpression of *Ptprn2*, *Tom1l2*, and *Pam* inhibited stimulated secretion of GLuc. The overexpression of *Stk39* increased AVP-GLuc secretion in the control condition, whereas stimulated secretion was blunted by the overexpression of *Ptprn2* and *Pam* (Fig. 6F). The phosphonull mutants for *Ptprn2*, *Tom1l2*, and *Pam*, unlike their wild-type forms, did not decrease GLuc secretion, suggesting that phosphorylation at these specific sites is important for controlling secretion (Fig. 6G). The *Pam* mutant was similarly not capable of inhibiting AVP-GLuc secretion (Fig. 6H). Whereas wild-type *Tom1l2* had no effect on AVP-GLuc secretion, the phosphonull mutant for *Tom1l2* stimulated control secretion. We further show that mutation of *Ptprn2* transformed it from an inhibitor to a stimulator of AVP-GLuc secretion in the stimulated condition. PTPRN2 is known to function in vesicle-mediated secretory processes and is expressed by nerve terminals in the posterior lobe of the pituitary (*32*). TOM1L2 is also known to function in vesicle trafficking in the synapse (*33*). The TOM1L2 and PTPRN2 phosphosites are increased in abundance after 14 days selectively in females, consistent with liraglutide-mediated sex-specific changes to AVP secretion.

### Liraglutide alters AVP-regulated signaling pathways in the rat kidney

**Fig. 7.**
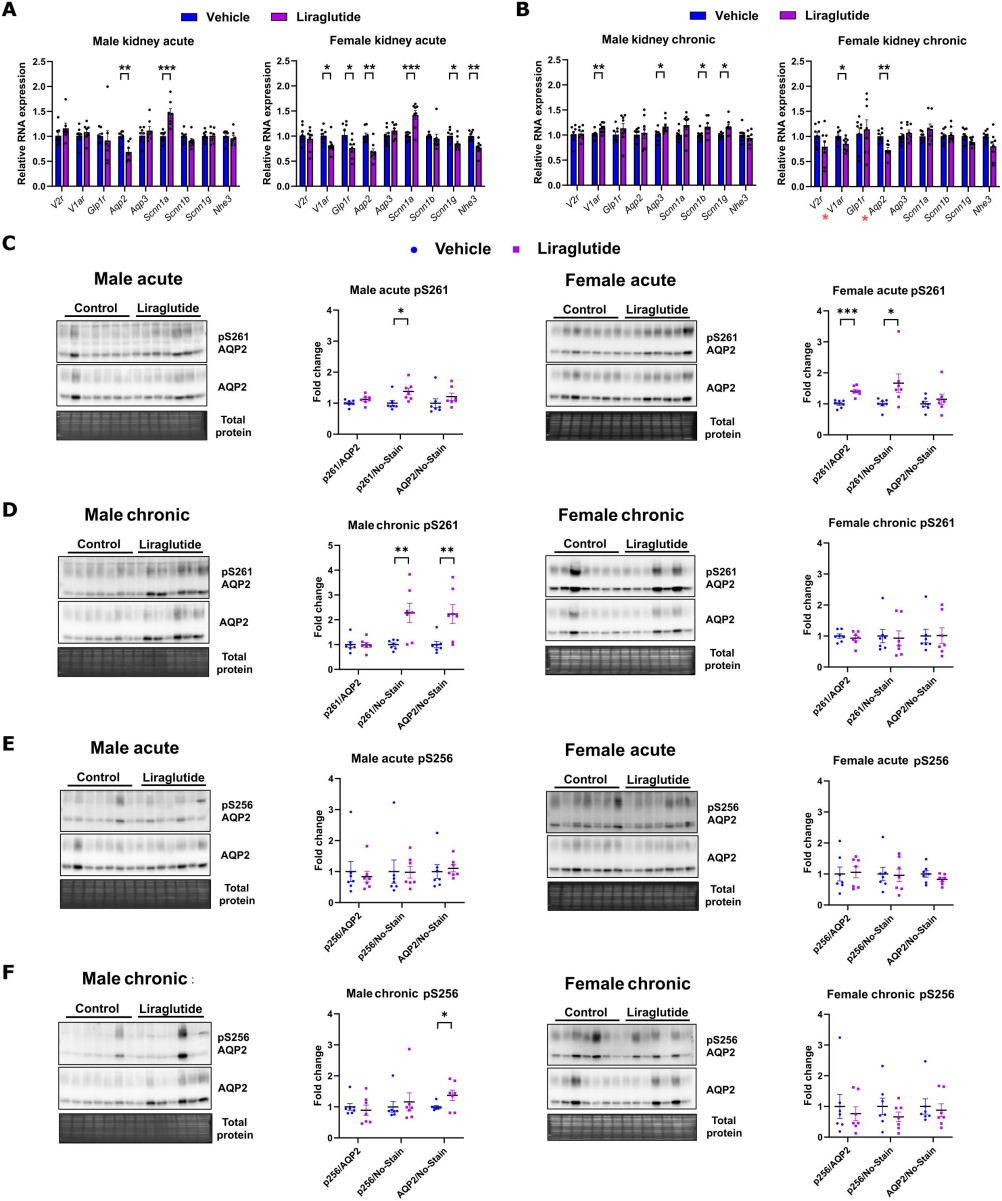
Effects of sex and duration of liraglutide treatment on the kidney fluid handling molecular mechanisms. Relative mRNA expression of selected genes involved in fluid handling investigated by qRT-PCR in the male and female kidney at 4 hours [(**A**); acute] and 14 days [(**B**); chronic] of liraglutide treatment. (**C**) Western blot of pS261 AQP2 immunoreactive bands in membrane fractions from kidneys of acute male and female control and liraglutide-treated rats. (**D**) Western blot of pS261 AQP2 immunoreactive bands in membrane fractions from kidneys of chronic male and female control and liraglutide-treated rats. (**E**) Western blot of pS256 AQP2 immunoreactive bands in membrane fractions from kidneys of acute male and female control and liraglutide-treated rats. (**F**) Western blot of pS256 AQP2 immunoreactive bands in membrane fractions from kidneys of chronic male and female control and liraglutide-treated rats. The total protein stain, No-Stain, and total AQP2 were used for the normalization of Western blotting data. Statistical analyses were performed by independent-sample unpaired *t* tests. Values are means ± SEM of *n* = 7 to 8 biological replicates per group. ^∗^*P* ≤ 0.05; ^∗∗^*P* ≤ 0.01; ^∗∗∗^*P* ≤ 0.001.

We asked about alterations to AVP signaling in the periphery, focusing our attention on the brain-kidney axis. We first looked at mRNA abundances of selected kidney gene targets guided by the literature. We identify significantly decreased expression of *Aqp2* and significantly increased expression of *Scnn1a* in both sexes at 4 hours (Fig. 7A). We additionally show decreased expression of *V1ar*, *Glp1r*, *Scnn1g*, and *Nhe3* in the female at 4 hours. In the male kidney, expression levels of *V1ar*, *Aqp3*, *Scnn1b*, and *Scnn1g* were increased after 14 days (Fig. 7B). The mRNA expression levels of *V1ar* and *Aqp2* remained significantly lower at 14 days in the female kidney. We then asked about the abundances of two key phosphosites in the membrane, pS256 and pS261, that are known to be altered by AVP, pS256 being increased by AVP and pS261 being decreased by AVP. For both sexes, pS261 was significantly increased at 4 hours by liraglutide treatment, but the ratio of p261/AQP2 was only significantly increased in females (Fig. 7C). In male 14-day kidney samples, the membrane content of pS261 and total AQP2 protein level increased, but the ratio of pS261/AQP2 did not, a response that was not found in females (Fig. 7D). We next investigated the expression of pS256 where we found no significant differences (Fig. 7, E and F). Thus, we identify AQP2 as a target for regulation by liraglutide, with sex-specific changes with more prolonged treatment with this drug.

## DISCUSSION

We designed and performed an acute clinical protocol to study the effects of liraglutide on AVP secretion in humans. We show that, acutely, liraglutide decreases plasma AVP levels in healthy humans. A mild salt challenge was successful in both subtly increasing plasma sodium and AVP levels. The translational confirmation for inhibitory actions of liraglutide on AVP secretion from rat to human raised questions about possible clinical benefits on renal and cardiovascular health or potential risks to these systems, when presented with this knowledge. Thus, we moved our attention to explore the effects of longer-term liraglutide treatment on the AVP system in our rat model, which is of greater clinical relevance as GLP-1RA is being used chronically in humans. We successfully created an assay to investigate the effects of specific proteins and protein phosphosites on AVP secretion. Investigation of the major downstream target for circulating AVP, the kidney, highlighted disturbances to crucial AVP-mediated downstream signaling pathways that control fluid homeostasis. All knowledge is important to better understand the known side effects, potential clinical benefits, and sex differences of GLP-1RA medications (*34*).

The influence of biological sex on the pharmacokinetics and pharmacodynamics of GLP-1RA treatment is important to consider in relation to altered AVP secretion and downstream signaling at AVP receptors (*35*). In humans and other species, males have more and larger AVP neurons, more AVP fibers, and altered V_1A_ receptor binding densities (*36*–*39*). Both the V_1A_ and V_1B_ receptors have been shown to be more sensitive to AVP in women compared to men, and women tend to have lower levels of plasma AVP (*40*–*43*). AVP sex differences have been associated with differences in behavior through actions in the brain (*44*). It has been shown that osmotic stimulation increases AVP release in the brain and periphery, whereas stressful stimuli can increase release only in the brain (*45*–*47*). We do not know whether release in the brain is being affected by GLP-1RA treatment, although we have previously described changes to neuronal activity that could infer this (*20*).

In relation to PP AVP secretion, we identify posttranslational changes to synaptic proteins that can explain disturbances to AVP secretion. We have previously identified that liraglutide treatment increases phosphorylation of soluble NSF attachment protein receptor complex protein SNAP25, which is instrumental in PP neuropeptide release (*20*). In the present study, this hyperphosphorylation at T138 persists following 14 days of treatment, but only in females. The longer-term phosphorylation of PTPRN2 and TOM1L2 by liraglutide in females provides an additional explanation for the reported sex differences in AVP secretion. In females, sex-specific differences in PTPRN2 have been described in the normal accumulation and secretion of anterior pituitary hormones (*48*), suggesting that this protein is an important sex-specific controller of hormone secretion. We further show different responses to liraglutide in the kidneys of males and females. Emerging evidence suggests sex differences in renal transporters and electrolyte homeostasis in kidney (*49*). Therefore, the sexually dimorphic patterns of AVP release, together with alterations in the PP and the kidney, likely collectively contribute to differing responses in males and females.

Although it is well-documented that GLP-1RA, including liraglutide, elicits diuresis and natriuresis (*50*–*52*), the mechanisms responsible have not been explored in detail. Our study suggests that AVP has a key role. The regulation of AQP2 by AVP is known to involve increased transcription of the mRNA and posttranslational modifications by phosphorylation as we observed (*53*–*57*). In isolated rat inner medullary collecting duct, treatment with AVP increases pS256, pS264, and pS269 but reduces pS261 phosphorylation (*58*). A decrease in pS261 phosphorylation is thought to increase the stability of the AQP2 protein without altering AQP2 trafficking, being associated with reduced AQP2 ubiquitylation and proteasomal degradation, although, notably, differences have been observed across studies (*59*–*61*). In contrast, pS261 phosphorylation leads to increased ubiquitylation of AQP2 and, ultimately, its degradation (*60*). It has been suggested that pS261 may be involved in the apical translocation of pS256 and pS269 (*62*). Thus, a decrease in AVP release may underpin changes to *Aqp2* mRNA abundance and AQP2 phosphorylation and, as such, contribute to GLP-1RA diuretic and natriuretic actions. However, other mechanisms could account for changes to AQP2 phosphorylation as adenosine 5′-triphosphate and dopamine have been found to prevent AVP-dependent water reabsorption through increased phosphorylation at pS261 (*63*). The observed sex differences in AQP2 could result from gonadal hormones as studies have shown that estrogen can regulate AQP2 expression in the kidney (*64*).

Increased plasma AVP levels have been associated with the pathophysiology of both diabetic kidney disease and chronic kidney disease (*65*, *66*), suggesting that decreasing circulating AVP as seen here with GLP-1RA may be clinically beneficial. Antagonists of the V_1A_ receptor have emerged as a promising therapeutic potential for the treatment of patients with chronic kidney disease in their own right (*67*–*69*). It is known that renal V_1A_ receptors play an important role in the regulation of systemic arterial blood pressure and renal medullary blood flow as well as urinary acidification. In the rat, the V_1A_ receptor agonist, Pmp^1^-Tyr(Me)^2^-vasopressin, administered in combination with GLP-1RA exenatide results in increased natriuresis in response to salt loading, suggesting a role for this receptor in sodium retention (*70*). Furthermore, in V_1A_ receptor knockout mice, research suggests that AVP regulates the RAAS via V_1A_ receptors in macula densa cells of the distal tubule to regulate body fluid homeostasis and glomerular filtration rate (*71*). As kidney protective actions of GLP-1RA are increasingly being explored, and semaglutide has received approval for the treatment of chronic kidney disease in patients with diabetes (*72*), alterations to the AVP system are important to consider in the clinical setting where GLP-1RA–modulated alterations to AVP secretion could be contributing to kidney and cardiovascular protection in patients.

We must address some limitations of this study. This study has been performed in healthy humans after a single injection. Understanding the actions of GLP-1RA in health and disease is becoming increasingly important as celebrity endorsements have caused enormous social media interest that continues to boost popular demand for cosmetic use of GLP-1RA in healthy individuals outside of the clinic. This study was not sufficiently powered to look for sex differences in humans. For proteomics studies, we minimized the postmortem sampling time to preserve phosphorylation events from drug treatment rather than post sampling (*73*). Thus, we acknowledge that samples contain intermediate lobe melanotrophs and pituicytes, the glial cells of the pituitary. In this study, we did not measure urine output, but we have previously described changes to urine parameters in the liraglutide-treated rat during water deprivation.

**Fig. 8.**
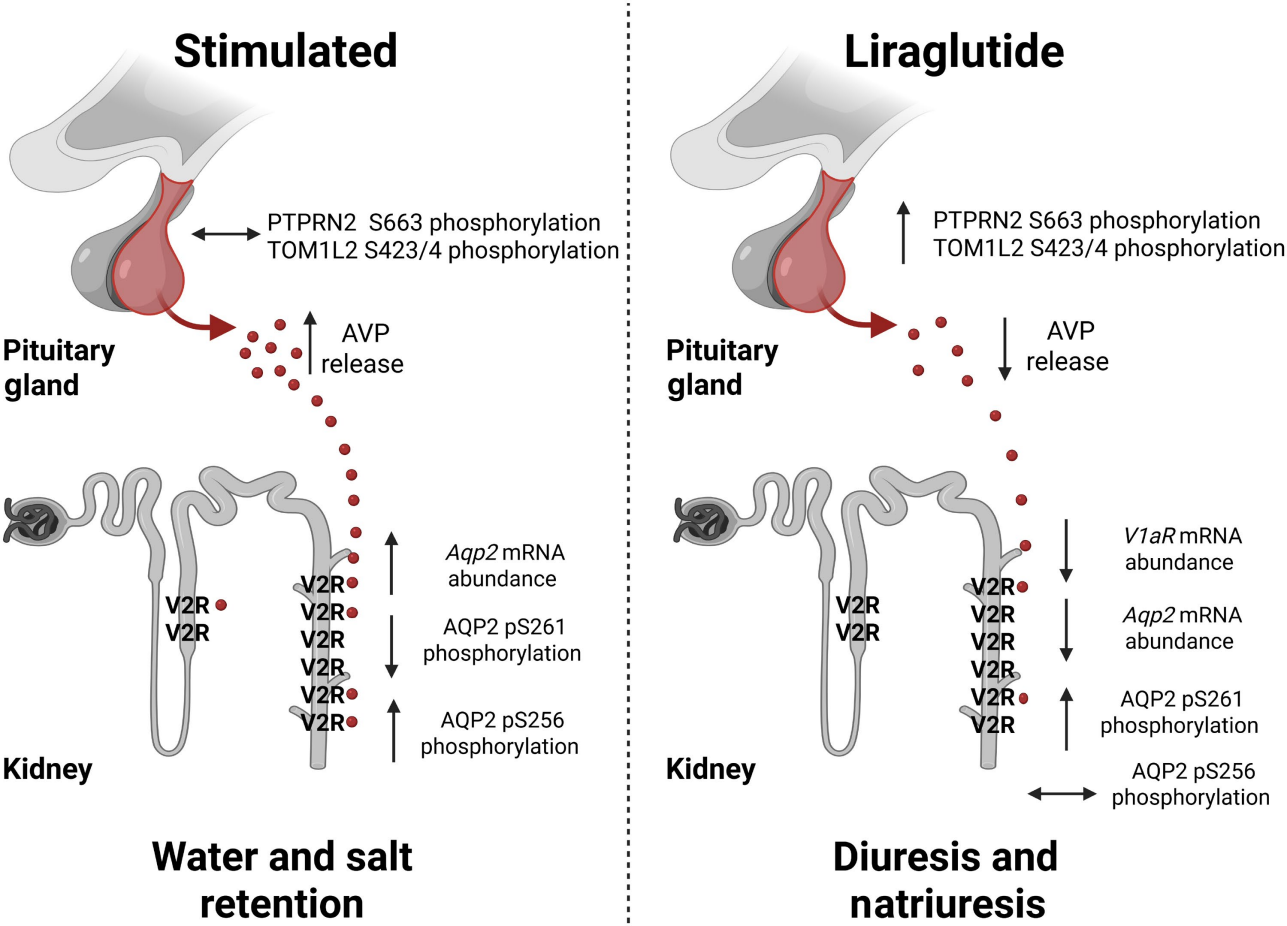
Summary of liraglutide effects on the AVP brain-kidney axis. Stimulation of AVP secretion, for example, by water deprivation, is known to increase renal vasopressin receptor activation leading to increased *Aqp2* transcription, decreased AQP2 pS261 phosphorylation, and increased AQP2 pS256 phosphorylation. This promotes increased water uptake, which is mediated by the translocation of AQP2 water channels to the apical membrane of collecting duct principal cells. We show that treatment with liraglutide acts to dampen this signaling axis. Liraglutide inhibits AVP release by altering the phosphorylation status of synaptic proteins PTPRN2 and TOM1L2 in the posterior lobe of the pituitary gland the site of storage and release. This alters renal AVP-mediated downstream signaling pathways, resulting in decreased *Aqp2* mRNA expression and increased AQP2 pS261 phosphorylation, both opposing actions to those reported by AVP stimulation. This will result in diuresis and natriuresis, which are two well-known responses to GLP-1RA treatment. Created in BioRender. M. Greenwood (2026), https://BioRender.com/o7gv7i5.

In summary, we have systematically investigated the AVP system from synthesis to storage to secretion in GLP-1RA–treated rats where we identified the PP, a known major site of GLP-1R expression in humans and rodents, as a major target for regulation of AVP secretion by liraglutide. We were able to develop assays to address the functional roles of synaptic protein phosphorylation on AVP secretion and identified alterations to downstream AVP signaling pathways and, ultimately, body fluid handling. Thus, our data suggest fundamental changes to the bodies’ key water homeostatic control system in the presence of long-acting GLP-1RA that will be presented, in part, by diuresis and natriuresis in patients (Fig. 8). This knowledge expands our understanding of fluid-regulatory hormones whose secretion is influenced by GLP-1RAs, including angiotensin II (*74*–*76*), aldosterone (*26*, *77*), and atrial natriuretic peptide (*50*, *52*). Thus, not only is water intake reduced because of decreased thirst sensation, but the capacity to conserve water is also diminished, at least acutely. Collectively, these findings suggest a complex interplay between GLP-1RA and the major hormone modulators of fluid balance in humans, which warrants further clinical investigation.

We should now also consider renoprotective and cardiovascular benefits of the described interactions of GLP-1RA with the AVP system in diseases associated with sustained increases in AVP secretion. These findings are of direct clinical relevance and may have implications for stratified risk assessment among patients particularly with fluid handling problems including the elderly (*78*, *79*). The knowledge of sex differences in hormone signaling may help with the development of better treatments that mitigate some of the contraindications in patients.

## MATERIALS AND METHODS

### Human

#### Study design

This study was a single center, open-label, before-and-after study of the feasibility of the detection of changes in hypothalamo-neurohypophysial system hormone release in healthy adults in response to GLP-1 receptor activation conducted in North Bristol NHS Trust, UK. The main inclusion criteria were as follows: 18 to 55 years of age; written and witnessed informed consent to participate; and body mass index between 18 and 30 kg/m^2^. The main exclusion criteria were as follows: breastfeeding and pregnancy; use of inadequate form of contraception for female participants; use of therapies that can affect fluid and water balance as assessed by the principal investigator; hypercalcemia found on screening; history of diabetes or abnormal HbA1c found on screening; history of renal failure or abnormal renal function found on screening; history of pituitary disorders; history of uncontrolled hypothyroidism or hyperthyroidism or abnormal thyroid function tests found on screening; and abnormal serum electrolytes found on screening.

Eighteen participants (11 females and 7 males) were recruited from staff at North Bristol NHs Trust from February to May 2024. After the screening, participants attended the first study visit. Participants attended the study center in the morning after having a light breakfast. They were asked to drink to thirst only, 24 hours before the study visit and up to the salt challenge point. They were offered an additional 250 ml of water (optional) at the beginning of the study visit to ensure no additional thirst drive at the start of the study. The salt challenge was achieved by oral intake of 5 g of sodium chloride dissolved in 100 ml of flavored water. After the salt challenge, participants were able to take small sips of water to keep their mouth moist and reduce sense of thirst, with the limit of 100 ml of water (in addition to water taken with salt) in the last 60 min of the procedure.

Samples were taken as follows: blood for sodium, potassium, urea, creatinine, chloride, serum osmolality, and plasma copeptin and AVP before the salt challenge (0 min, 15 min, 30 min, 1 hour, and 3 hours of the study) and 15 min, 30 min, and 1 hour after the salt challenge; blood for glucose at 0, 3, and 4 hours of study; and urine for urine osmolality, sodium, and potassium before salt challenge (0 and 3 hours of the study) and 1 hour after the salt challenge (4 hours of the study). The second study visit was carried out 1 to 4 weeks after the first visit under the same conditions and according to the same schedule as stated above with addition of administration of a single dose of 1.2-mg liraglutide, subcutaneously into the abdomen, after taking a baseline set of samples (time point 0).

#### Plasma and hormone measures

Biochemistry tests analyzed on each collected sample were by the following methods on the AU5800 analytical platform (Beckman Coulter Inc., UK): serum and urine sodium, potassium, and chloride by ion-selective electrode; serum creatinine by enzymatic colorimetry via sarcosine oxidase; serum urea by enzymatic colorimetry via urease; and serum glucose by enzymatic hexokinase. Serum and urine osmolality was analyzed by freeze point depression on the Advanced Micro-osmometer 3320 (Advanced Instruments, Massachusetts, USA). Samples were analyzed on the day of collection, stored at 4°C for 72 hours, and discarded in line with routine clinical laboratory processes. All analysis was conducted in a UK clinical biochemistry lab with active United Kingdom Accreditation Service accreditation against ISO15189:2012 during the study period (now accredited against ISO15189:2022 at time of manuscript). All analyzers were calibrated and had acceptable internal quality control at the time of analysis for each study day.

Blood samples for hormone measures (AVP, copeptin, and cortisol) were collected in 10-ml BD Vacutainer EDTA tubes chilled in an iced water bath, and, within 30 min of collection, tubes were centrifuged at 1600*g* for 20 min at 4°C. Plasma was dispensed into 1-ml aliquots in tubes containing 500 kallikrein inhibitor units (KIU) of the protease inhibitor aprotinin (Selleck Chemicals, S7377-SEL) and stored at −40°C for up to 5 hours before being transferred on dry ice for longer-term storage at −80°C. Human plasma hormone measures—AVP, copeptin, and cortisol—were determined by enzyme-linked immunosorbent assay (ELISA) in accordance with manufacturer’s protocols (AVP, Enzo Life Sciences, catalog no. ADI-900-017A; copeptin, Cloud-Clone Corp, SEA365Hu; and cortisol, R&D Systems, catalog no. KGE008B). AVP was extracted from 1.5 ml of plasma by the acetone/petroleum ether method according to the manufacturer’s protocols. Samples were dried using a nitrogen gas blower before being reconstituted by vortexing in assay buffer in accordance with manufacturer’s protocols. The 16 samples collected for each participant across visits 1 and 2 were processed together and assayed on the same ELISA plate for each hormone to reduce between-run imprecision. All samples were assayed in duplicate, and the signal was detected on an iMark microplate absorbance reader (Bio-Rad Laboratories).

### Animals

Our study examined male and female animals, and sex-dimorphic effects are reported. Animal sample sizes were calculated by making an estimate of variability from previous experiments that we have performed with two groups, using similar approaches, under similar conditions, in rats (*20*). These data provide an estimate of the expected SD of the primary outcome, and, then, power calculations in GPower 3.1 have been used to calculate the sample size for the experimental group (*80*). All studies were performed with time-matched experimental controls run in parallel and sampled on the same day. For animal studies with two groups, randomization was performed by the flip of a coin to determine which group the animal was assigned. Animals to be injected were handled daily for 1 week at the time of injection.

Taking note of the consensus in the scientific community that sex is a significant biologic variable and known sex differences in the control of fluid homeostasis (*81*), we included equal numbers for both sexes in all rat studies. Male and female Sprague-Dawley rats weighing 225 to 249 g and 175 to 199 g, respectively, were purchased from commercial supplier Envigo (RRID: 70508). Rats were housed under a 12-hour light/12-hour dark cycle at a temperature of 21° to 22°C and a relative humidity of 40 to 50% with food and water ad libitum. Through the University of Bristol Animal Management Information System (AMIS), animals are randomly assigned to cages by Animal Services Unit staff. The number of cages and the number of animals per cage are preset by the investigator on AMIS, but the allocation is independently performed. Rats were singly housed in Tecniplast 1290 conventional rat cages for 2-week period of acclimation before experimentation. Cages contained sawdust, bedding material, cardboard tubes, and wooden chews for enrichment. Measures of food and water, and animals were performed by weight. The bottle was removed to measure food intake, and the cage lid containing the food was removed to measure food intake. Nighttime measures were performed under dim red lighting.

#### GLP-1RA studies

Liraglutide (Tocris, 6517/1) was prepared in normal saline to a concentration of 100 μg/ml. Injections of 100 μg/kg body weight or normal saline vehicle were performed intraperitoneally using a 30-gauge insulin syringe. The dose of liraglutide and route of administration has previously been described in the rat (*12*, *82*, *83*).

#### GLP-1RA study series 1—Liraglutide 4 hours

Animals were housed singly for 4 weeks before experimentation. Injections were performed during the final 30 min of the light phase before the lights went off. Animals were euthanized 4 to 5 hours after injection of liraglutide or vehicle.

#### GLP-1RA study series 2—Liraglutide 14 days

Animals were singly housed for 2 weeks before experimentation. Injections were performed daily for 14 days during the final 30 min of the light phase before the lights went off. Animals were euthanized 4 to 5 hours after the final injection of liraglutide or vehicle. On days 1, 6, and 12, measures of food and water intake were recorded at three timed intervals of 0 to 4, 4 to 8, and 8 to 24 hours following injection.

#### Rat sampling

Rats were humanely euthanized by striking the cranium and then immediately decapitated with a small animal guillotine (Harvard Apparatus). Trunk blood was collected in 4-ml BD Vacutainer EDTA tubes (BD, 368860) containing 500 KIU of the protease inhibitor aprotinin (MERCK, 10236624001) chilled in an iced water bath and within 1 hour of collection centrifuged at 1600*g* for 20 min at 4°C (*20*). Plasma was collected in 1-ml aliquots and snap frozen in liquid nitrogen and stored at −80°C. Brains were rapidly removed from the cranium and placed into a chilled rodent brain matrix (ASI Instruments, RBM-4000c) on ice for separation of the fore and hind brain regions. The brain was placed cut edge down onto aluminum foil resting on pellets of dry ice and immediately covered with powdered dry ice (within 3 min of decapitation). Brains were wrapped in foil and stored at −80°C. Pituitaries were placed in 0.5-ml tubes and placed on dry ice before storage at −80°C. Kidneys were removed and wrapped in aluminum foil before being submerged in liquid nitrogen and stored at −80°C.

#### Isolation of RNA and real-time qRT-PCR

The collection and processing of brains by punching has previously been described (*84*–*86*). Frozen kidney was ground to a fine powder using a pestle and mortar and processed as described previously (*87*). Total RNA was extracted from kidney and brain samples using a QIAGEN RNeasy Mini kit (QIAGEN, 74104), as previously described (*84*–*86*). cDNA synthesis and qRT-PCRs were carried out as described previously (*88*). For relative quantification of gene expression, the 2^−ΔΔCT^ method was used (*89*). The internal control gene used for SON and PVN was the reference gene *Rpl19* (*20*, *84*–*86*, *88*, *90*). We tested five candidate reference genes for the kidney, *Sdha*, *Actin*, *Gapdh*, *Pgk1*, and *Ppia* in this study and *Gapdh* ranked as the top reference gene using the computational tools in RefFinder (*91*). All qRT-PCR primer sequences can be found in table S3.

#### Plasma measures

##### Rat plasma hormone measures

Copeptin and corticosterone were determined in plasma by ELISA (copeptin, Cloud-Clone Corp, SEA365Ra; and corticosterone, ALPCO, 55-CORMS-E01). Corticosterone levels below the detection threshold of the assay were given the lowest detectable value. Pituitary hormone measures were performed as described previously (*85*) by AVP ELISA (AVP, Enzo Life Sciences, catalog no. ADI-900-017A). To measure PVN and SON AVP content, punch samples were resuspended in 500 μl of ice-cold 10 mM HCl and immediately sonicated for 15 s before incubation at 85°C for 20 min. Cellular debris was removed by centrifugation at 3000*g* for 30 min at 4°C. The supernatant was diluted (1:62.5) with assay buffer, and ELISAs were performed following the manufacturer’s protocol. All samples were assayed in duplicate with male and female samples on separate plates, and the signal was detected on an iMark microplate absorbance reader (Bio-Rad Laboratories).

#### Proteomics and phosphoproteomics

The proteomic sample processing pipeline for NIL samples has previously been described in detail (*92*). We performed two separate analyses: one to compare the total protein levels across the samples and a second in which we included a phosphopeptide enrichment step to allow us to compare phosphopeptide levels across the same samples. As such, the normalization factor for the total protein dataset was applied to the phospho dataset as it provided a more useful normalization metric than total phosphopeptide abundance, which is more likely to vary on the basis of the biological conditions of interest. Where possible, phosphopeptides were matched to corresponding proteins in the total protein dataset, and the log_2_-normalized protein abundance was subtracted from the log_2_-normalized phosphopeptide abundance to adjust the phosphopeptide abundance for any changes in the total protein level. Statistical significance was then determined using Welch’s *t* tests between the conditions of interest. Because it has been discussed that the use of multiple testing–corrected false discovery rate (FDR) maybe too blunt and restrictive for proteomic analysis (*26*), especially when analyzing such a heterogeneous and complex tissue as the brain (*27*–*32*), we considered uncorrected *P* < 0.05 as differentially produced proteins and phosphosites in our NIL analysis. Proteomic datasets can be found in table S4.

#### Gene Ontology and pathways analysis

Overlap analyses of differentially expressed proteins and phosphoproteins were performed using VENNY 2.1.0. Proteomics gene ontologies were performed using SynGO (*93*). In SynGO, we used brain-expressed genes as the background list, and terms were identified as significant if they were enriched at 1% FDR.

#### Western blotting

To prepare crude kidney membrane protein fractions, we added 2 ml of ice-cold extraction buffer (pH 7.6) containing 10 mM triethanolamine, 250 mM sucrose, 1 mM phenylmethylsulfonyl fluoride, pierce protease inhibitor cocktail (Thermo Fisher Scientific, A32963), and phosphatase inhibitor cocktail (Thermo Fisher Scientific, A32957) to ∼200 mg of powdered kidney tissue and vortexed rigorously. Samples were maintained on ice for an additional 30 min, vortexing every 10 min. To remove cellular debris, samples were centrifuged at 1000*g* for 10 min at 4°C. The supernatant was transferred to a fresh tube and centrifuged at 17,000*g* for 20 min to achieve an enriched protein membrane pellet. The supernatant was discarded and the pellet washed two times with extraction buffer and then resuspended in radioimmunoprecipitation assay buffer and transferred to a fresh tube (*20*). The samples were stored at −80°C.

Protein samples were prepared to 1× Laemmli buffer solution [2% (w/v) SDS, 10% glycerol, 5% (v/v) 2-mercaptoethanol, 0.002% (v/v) bromophenol blue, and 0.125 M tris-HCl (pH 6.8)]. The kidney protein samples were heated at 60°C for 20 min, whereas the NIL protein samples were heated at 95°C for 10 min. For semiquantitative analysis of protein levels, 20 μg/lane of total protein per lane was loaded for control and experimental samples. Proteins were fractionated on SDS polyacrylamide gels and transferred to Immobilon-P PVDF Membrane (MERCK, IPVH00010). For molecular weight estimation, one lane on each blot was loaded with Novex Sharp Pre-Stained Protein Standard (Life Technologies, LC5800). After transfer, the No-Stain Protein Labeling Reagent (Thermo Fisher Scientific, A44717) was applied to the membrane following the manufacturer’s protocols to allow for total protein normalization of Western blots.

Membranes were blocked by incubation in 5% (w/v) skimmed in tris-buffered saline [150 mM NaCl and 20 mM tris-HCl (pH 7.6)] with 0.1% (v/v) Tween 20 (TBST) for 1 hour. All primary antibody incubations were performed in 5% (w/v) bovine serum albumin (Sigma-Aldrich, A7906) prepared in TBST: anti-AQP2 (E-2) (1:1000; Santa Cruz Biotechnology, sc-515770, RRID:AB_2810957), anti-AQP2 (phospho Ser^261^) antibody (1:2500; PhosphoSolutions, p112-261, RRID:AB_2492041), anti-AQP2 (phospho S256) antibody (1:2000; Abcam ab111346, RRID:AB_10887744), and anti-S100A9 antibody (1:2000; R&D Systems, AF2065, RRID:AB_2184263). After overnight incubation at 4°C with gentle rocking, membranes were washed three times for 10 min each with TBST. Membranes were incubated for 1 hour with secondary antibodies, anti-rabbit (Sigma-Aldrich, A0545, RRID:AB_257896), anti-mouse (Sigma-Aldrich, A9044, RRID:AB_258431), or anti-goat (Sigma-Aldrich, catalog no. A5420, RRID:AB_258242) conjugated with horseradish peroxidase, prepared in the primary antibody dilution buffer (1:20,000) with gentle rocking. Membranes were washed three times for 10 min each with TBST. The signal was detected by chemiluminescence SuperSignal West Dura Extended Duration Substrate for other antibodies (Thermo Fisher Scientific, 34075) using a Syngene G:Box imaging system. Immunoblots were stripped using ReBlot Plus Strong Antibody Stripping Solution (MERCK, 2504) after probing for phosphorylated AQP2 before assessing total AQP2 abundance. Band intensities were determined using Image Lab 6.0.1 (Bio-Rad, Hercules, CA, USA).

#### Cells and treatments

INS-1 832/13 Rat Insulinoma Cell Line (MERCK, SCC207, RRID:CVCL_7226) was cultured in RPMI-1640 (Sigma-Aldrich, R0883) supplemented with 2 mM l-glutamine (Sigma-Aldrich, TMS-002-C), 1 mM sodium pyruvate (Sigma-Aldrich, TMS-005-B), 10 mM Hepes (Sigma-Aldrich, TMS-003-C), 0.05 mM β-mercaptoethanol (Sigma-Aldrich, ES-007-E), 10% (v/v) fetal bovine serum (Sigma-Aldrich, ES-009-B), and penicillin-streptomycin (100 U/ml; Thermo Fisher Scientific, 15-140-163). Cells were incubated at 37°C in a humidified incubator with 5% (v/v) CO_2_. For secretion luciferase assays, we generated a fusion construct of the rat AVP prohormone cDNA with Gaussia luciferase cDNA (AVP-GLuc) containing the flexible linker sequence GGGGS by overlap extension PCR. A Gaussia luciferase plasmid was used as a control reference (pCMV-Gaussia-Dura Luc; Thermo Fisher Scientific, 16191). The amplified products were restriction digested and cloned into compatible restriction sites of pcDNA3.1^+^ plasmid. For overexpression constructs, *Ptprn2*, *Tom1l2*, *Syt7*, *Stk39*, *Scg6*, and *Pam* were amplified from rat SON cDNA, restriction digested, and cloned into compatible sites of pcDNA3.1^+^. Phosphonull mutants were generated by overlap extension PCR. All cDNAs were amplified using Phusion High-Fidelity DNA Polymerase (New England Biolabs). See table S3 for primer details.

For secretion assays, 4 × 10^4^ cells per well cells were seeded into 96-well white cell culture plates (Thermo Fisher Scientific, 10072151). After 16 hours, transfections were performed (90 ng pcDNA3.1 overexpression plasmid and 10 ng of Gaussia luciferase (GLuc or AVP-GLuc) plasmid per well) using Lipofectamine LTX transfection reagent (Thermo Fisher Scientific, 15338030). Forty-eight hours posttransfection cells were washed twice with 200 μl of Krebs Ringer Buffer secretion medium [120 mM NaCl, 5 mM KCl, 1 mM MgCl_2_, 2 mM CaCl_2_, 24 mM NaHCO_3_, 15 mM Hepes (pH 7.4), 5 mM glucose and bovine serum albumin (1 mg/ml) (Sigma-Aldrich, A7906)]. Cells were then incubated in secretion medium or secretion medium supplemented with 10 μM forskolin (Sigma-Aldrich, F6886) and 1 μM PMA (Sigma-Aldrich, P8139) for 1 hour. A 50-μl aliquot of medium was removed and transferred to a 96-well white plate, and the remaining medium was discarded. To the cells in the well, 20 μl of Passive Lysis Buffer was added, and plates were placed on an orbital shaker with gentle rocking for 15 min at room temperature. To the 50-μl aliquots, 50 μl of Gaussia assay buffer containing 10 μM coelenterazine (Apex-Bio, B6010) was added using a multichannel pipette, and plates were immediately measured as described for a similar system to measure insulin secretion (*94*). To measure cell Gaussia luciferase content, the Renilla Luciferase Assay System was used (Promega, E2820). Luciferase measures were performed using a Promega GloMax 96 microplate luminometer.

#### Immunofluorescent staining

Immunofluorescent staining was performed as described previously (*84*, *90*). The primary antibodies used were GLP-1R (Abcam, ab218532, RRID:AB_2864762) and neurophysin I (PS41, RRID: AB_2313960). Images were captured using a Leica SP5-II AOBS confocal laser scanning microscope attached to a Leica DMI 6000 inverted epifluorescence microscope and Leica LAS-X acquisition software.

### Statistical analysis

Image brightness and contrast adjustments were made to the whole image in Leica LAS X software. Statistical analyses and plotting of data were performed in GraphPad Prism version 10. Statistical differences between two experimental groups were evaluated using independent-sample unpaired *t* tests. To analyze human serum electrolytes, a mixed-effects analysis of variance (ANOVA) was use because of some missing values. Two-way ANOVA with Fisher’s least significant difference post hoc test was used for planned comparisons where it was not sensible to have formal corrections for multiple comparisons. Two-way ANOVA with a Šídák’s post hoc test was used to compare control and experimental values at each time point where it was sensible to have formal corrections for multiple comparisons. The statistical tests for comparisons have been indicated in the figure legend text. Statistical analysis of qRT-PCR data was performed using the delta cycle threshold values. A Grubbs’ test was performed in GraphPad to identify any significant outliers with an α = 0.05. Data are presented as the means ± SEM where *P* ≤ 0.05 was considered statistically significant.

### Study approval

The study was carried out with the approval of the UK Research Ethics Service North West Liverpool Central Research Ethics Committee (reference 23/NW/0106). Written informed consent was obtained from all participants. The trial was conducted in compliance with the principles of the Declaration of Helsinki (2013) and the principles of good clinical practice and in accordance with all applicable regulatory requirements. All animal experiments were performed under Home Office UK licenses 30/3278 and PP9294977 held under and in strict accordance with the provisions of the UK Animals (Scientific Procedures) Act (1986); they had also been approved by the University of Bristol Animal Welfare and Ethical Review Board.
