# Supplementary material for: A clinical and experimental investigation of liraglutide effects on the brain-kidney axis
Source: Sci Adv. 2026 Aug 5;12(32):eaef4951. doi: 10.1126/sciadv.aef4951 (PMC13440386; doi:10.1126/sciadv.aef4951)
Supplement: Supplementary file 1 [file sciadv.aef4951_sm.pdf]

Science Advances
AAAS

Supplementary Materials for

A clinical and experimental investigation of liraglutide effects on the brain-kidney axis

Michael P. Greenwood *et al.*

Corresponding author: Michael P. Greenwood, mike.greenwood@bristol.ac.uk

Sci. Adv. **12**, eaef4951 (2026)
DOI: 10.1126/sciadv.aef4951

The PDF file includes:

Tables S1 to S3
Legend for table S4
Figs. S1 to S5

Other Supplementary Material for this manuscript includes the following:

Table S4

Supplemental Material

	Mean	SE	Median	Minimum	Maximum
Age (yr)	35.25	2.20	34.5	22	54
Height (m)	1.71	0.02	1.7	1.62	1.82
Weight (kg)	71.5	2.88	69.65	53.5	93
BMI (kg/m2)	24.63	0.86	25.3	19.2	30

Table S1. Baseline characteristics of study participants. SE, standard error.

	Visit 1					Visit 2				
	Mean	SE	Median	Minimum	Maximum	Mean	SE	Median	Minimum	Maximum
Osmolality (mOsmol/kg)	289.9	1.13	291	279	297	288.64	0.98	289	282	297
Sodium (mM)	139.6	0.46	140	136	143	139.07	0.23	139	138	140
Potassium (mM)	4.14	0.055	4.1	3.8	4.5	4.09	0.055	4.1	3.7	4.4
Glucose (mM)	4.56	0.25	4.5	2.6	6.8	4.75	0.15	4.6	3.6	5.9
Urine osmolality (mOsmol/kg)	689.3	66.95	796	102	1030	593.5	70.96	667	100	939
Urine sodium (mM)	101.1	12.84	89	25	195	79.64	12.91	73.5	13	192
Urine potassium (mM)	67.5	9.12	62	20	120	68.35	10.53	49.5	15	146
AVP (pg/ml)	4.83	0.69	3.37	2.27	12.08	5.14	0.56	4.54	2.57	10.21
Copeptin (pg/ml)	28.61	3.43	27.70	10.73	63.45	26.53	2.89	25.43	7.75	48.75
Cortisol (ng/ml)	148.9	12.98	148.9	83.28	297.2	154.01	15.65	146	101	361.9

Table S2. Baseline blood and urine measures on visits 1 and 2. SE, standard error.

Gene	Forward primer	Reverse primer
**qRT-PCR primers**		
Glp1r	GTTCCGCTGCTGTTCGTTAT	GCAAGCGTATGATGAGCCAA
c-Fos	AGCATGGGCTCCCCTGTCA	GAGACCAGAGTGGGCTGCA
hnAvp	GAGGCAAGAGGGCCACATC	CTCTCCTAGCCCATGACCCTT
Avp	TGCCTGCTACTTCCAGAACTGC	AGGGGAGACACTGTCTCAGCTC
Crh	CTCTCTGGATCTCACCTTCCAC	CTAAATGCAGAATCGTTTTGGC
Pgk1	TCCGCATCTCCATTTGGTGT	TCCCGATGCAGTAAAGACGA
Ppia	TTGCTGCAGACATGGTCAAC	CTTTCTCCCCAGTGCTCAGA
Actin	CGTAGCCATCCAGGCTGTGT	CCAGTGGTACGACCAGAGGC
Gapdh	ATGACTCTACCCACGGCAAG	CTGGAAGATGGTGATGGGTT
Rpl19	GCGTCTGCAGCCATGAGTA	TGGCATTGGCGATTTCGTTG
Sdha	GGCGGGATTCCCACTAACTA	ACAGACCAGGCACAATCTGA
Aqp2	GGGACTGTGCTTAGTGCATC	TGCAAACTCTGTTCGTGAGG
Scnn1a	TGTCGGAAGCCTTGTAGTGT	GCAAGGACAGCATCTCGAAG
Scnn1b	TCTCCTGGTGATAGGCCACT	AATGCCAACTAGGGCAAGGT
Scnn1g	CAGTGTGCAAGCAATCCTGT	ACCATTTCTCGGAAGCCTCA
Aqp3	TTGGACCTCGCCTTTTCACT	ACGAAGACACCACCAATGGA
Nhe3	CGCACTTCGACTGTCACTTT	GCTTTTGGAATGAGCGCCTA
V2r	CCCACACCCATAGATGACCT	GGGACCTGTTCCCAAAAATGT
V1ar	ATGCTGGGACATCACCTACC	GCATATAGGCAGACGCGAAC
**Cloning primers**		
AVP_GLuc	CCCAAGCTTGCCACCATGCTCGCCATGATGCTCAACAC	GTAGACCCGGGGCTTGGCAGAATC
GLuc linker	GATTCTGCCAAGCCCCGGGTCTACGGAGGAGGAGGATCAAAGCCCACCGAGAACAACGAGGA	CTAGTCTAGATTAGTCGCCGCCGGCGCCCTTG
Pam	CCCAAGCTTGCCACCATGGCCGGACGCGCCCGCAGCGGTCT	CTAGTCTAGATCAGGAGGAAGGTGCAGGCTTGGGCAG
Ptprn2	CCGGAATTCGCCACCATGGGGCTACCGCTCCCGCTTTT	CTAGTCTAGACTACTGGGGAAGGGCCTTCAGGAT
Tom1l2	CCCAAGCTTGCCACCATGGAGTTCCTGCTGGGGAATCCGTTCA	CTAGTCTAGATCACAGAGCAAAGAGCGCATCCTCAGA
Sty7	CCCAAGCTTGCCACCATGTACCGGGACCCGGAGGCGGCCA	CTAGTCTAGATCAGGCTTTCAGCTGGTGCCACTG
Scg6	CCCAAGCTTGCCACCATGGATCGCAGGTCCCGGGCTCA	CTAGTCTAGATTAGTGACGCCGGATGGGGATG
Stk39	CCGGAATTCGCCACCATGGCGGAGCCGAGCGGCTCG	CTAGTCTAGATCAGCTCACACTCAACTGGGCGAAC
**Phosphonull primers**		
Pam	AGATTCCGAGGAAAGGGAGCAGGCGGCTTAAATCTGGGA	TCCCAGATTTAAGCCGCCTGCTCCCTTTCCTCGGAATCT
Ptprn2	AAGCTGAAGGAGAAATTGGCAGGACTAGGAGCTGACCCC	GGGGTCAGCTCCTAGTCCTGCCAATTTCTCCTTCAGCTT
Tom1l2	CTAGACAATCGGAAACAGGCAGCAGAAATGAAGGGTGACGAC	GTCGTCACCCTTCATTTCTGCTGCCTGTTTCCGATTGTCTAG
Syt7	AACTCCTTGGAGACGGTGGGCGCACCAGACGCAGGGCGTGGGCGCGGTGAGAAG	CTTCTCACCGCGCCCACGCCCTGCGTCTGGTGCGCCCACCGTCTCCAAGGAGTT
Scg6	CCACCACTCAGGAGCCTGAGGCAGCAAGCGAGGGGGAGGAGCC	GGCTCCTCCCCCTCGCTTGCTGCCTCAGGCTCCTGAGTGGTGG
Stk39	TCAAGCGGTCACCTTCACAAGGCAGAAGATGGCGACTGGGAGTGG	CCACTCCCAGTCGCCATCTTCTGCCTTGTGAAGGTGACCGCTTGA

Table S3. Oligonucleotides used in this study.

Table S4. Proteomic data excel file. See ProteomeXchange Consortium via the PRIDE partner repository with unique dataset identifier PXD068252.

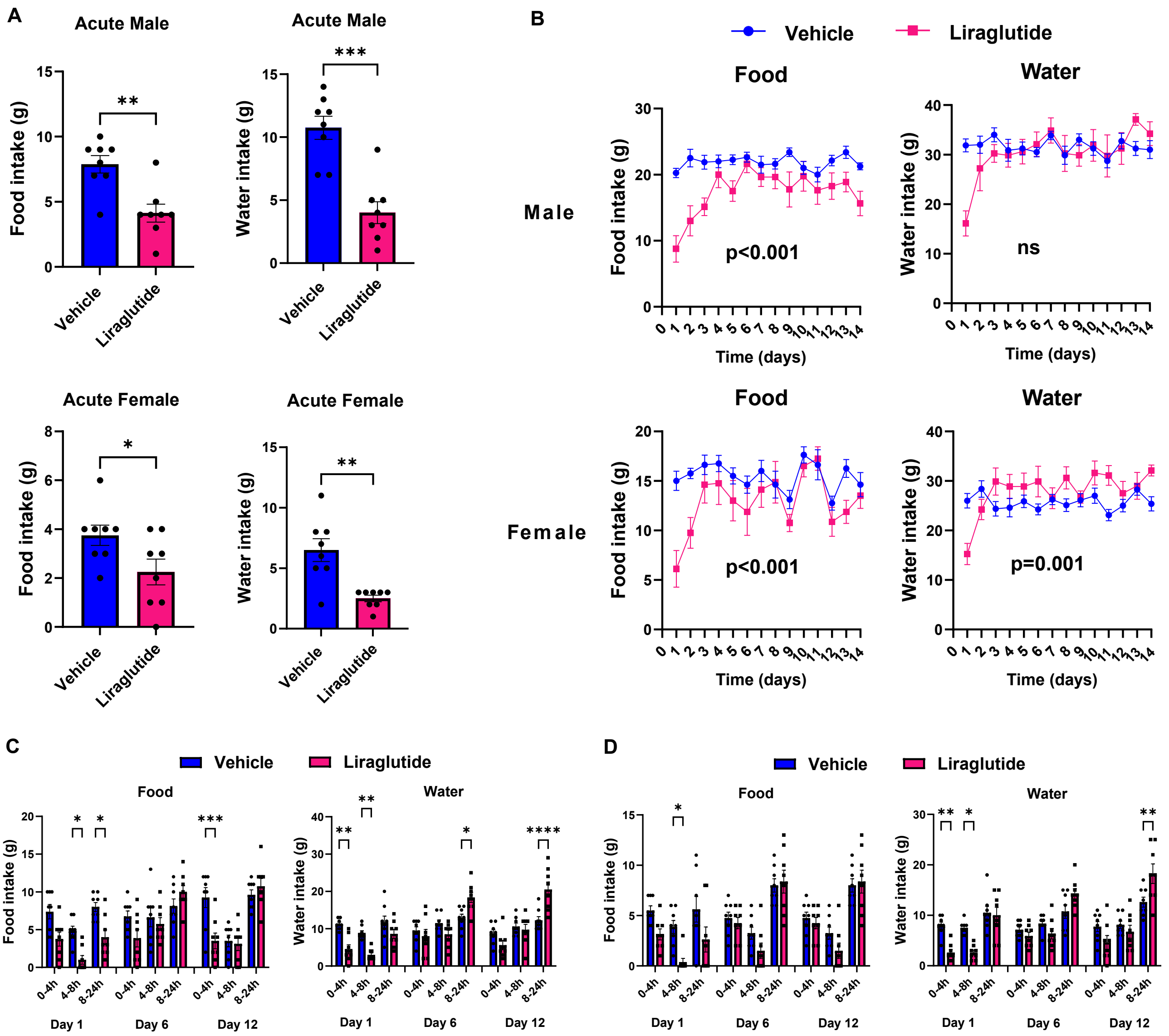

Figure S1. Dynamic fluctuations in food and water intake following GLP-1RA liraglutide treatment in the rat. **A**, endpoint measures of food intake and water intake in the 4-hour liraglutide treatment groups (acute). **B**, daily monitoring of food and water intake for the 14-day liraglutide treatment groups (chronic). **C** (male), **D** (female), measures of food and water intake separated into intervals of 0-4, 4-8, and 8-24 hours on days 1, 6, and 12 of liraglutide treatment. Statistical analyses were performed by independent-sample unpaired t tests **(A)**, repeated measures two-way ANOVA **(B)**, and two-way ANOVA with Sidak's post hoc test **(C, D)**. Values are means ± SEM of n = 8 animals per group. $*p \leq 0.05$, $**p \leq 0.01$, $***p \leq 0.001$.

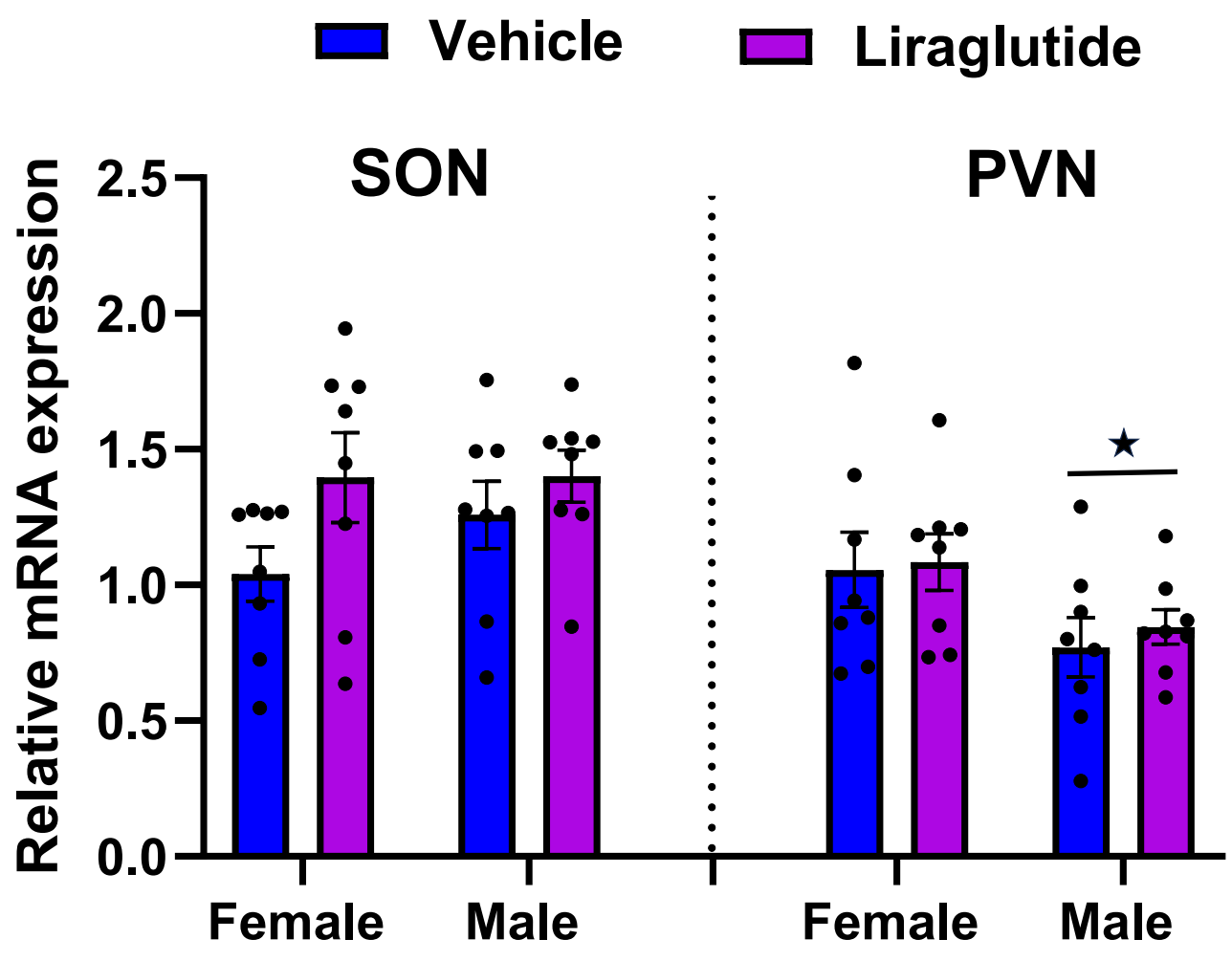

Figure S2. Sex differences in *Glp1r* expression. Relative mRNA expression of *Glp1r* in the SONs and PVNs in control and 14-day liraglutide-treated animals normalised to female control. Two-way ANOVA indicated significantly lower *Glp1r* expression ($F(1,28) = 5.964$, $p = 0.021$) in males compared to females. Values are means ± SEM of n = 8 animals per group. $*p \leq 0.05$.

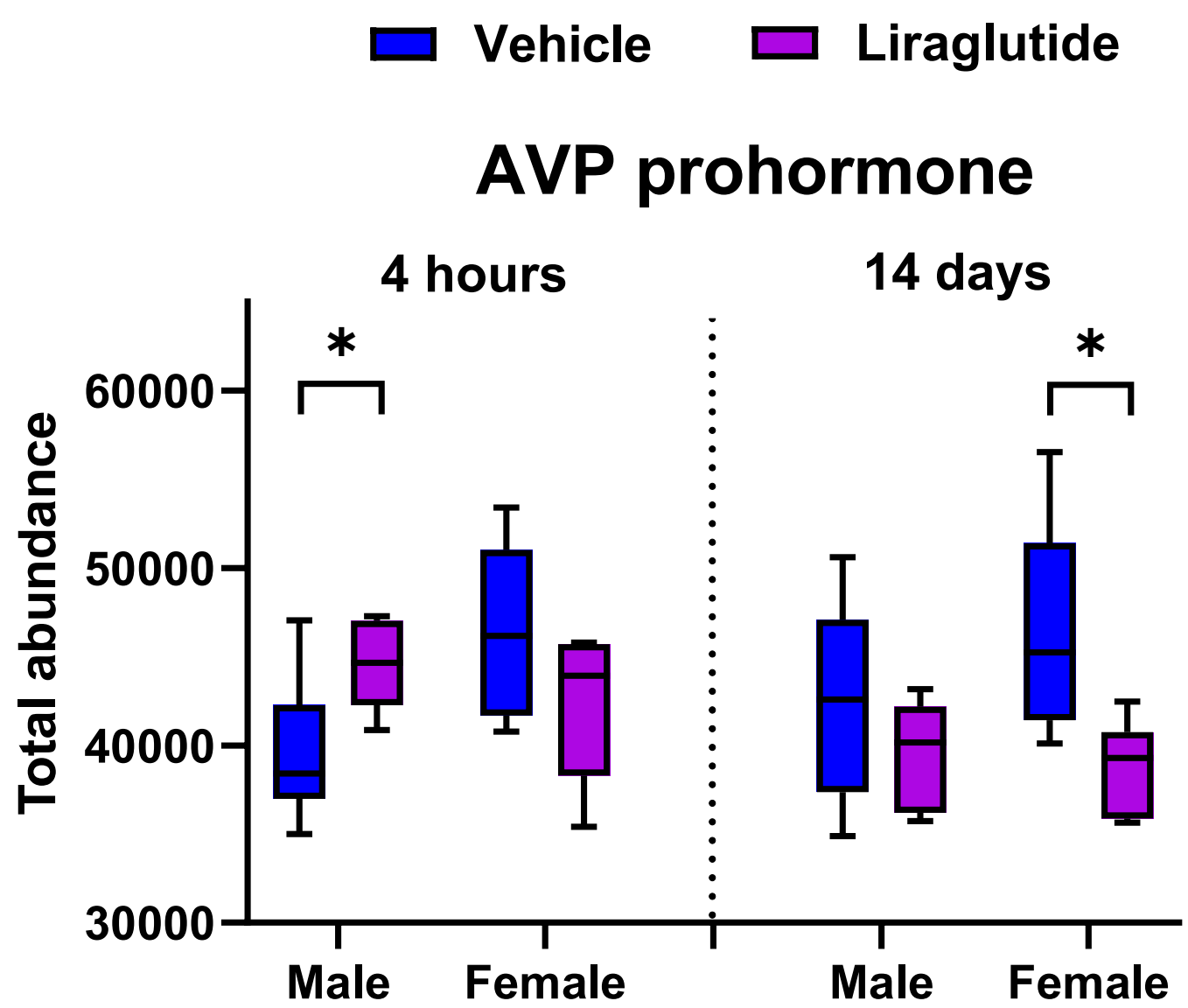

Figure S3. Proteomic data for AVP prohormone abundance in neurointermediate lobe samples of acute (4 hours) and chronic (14 days) male and female control and liraglutide-treated rats. $*p \leq 0.05$.

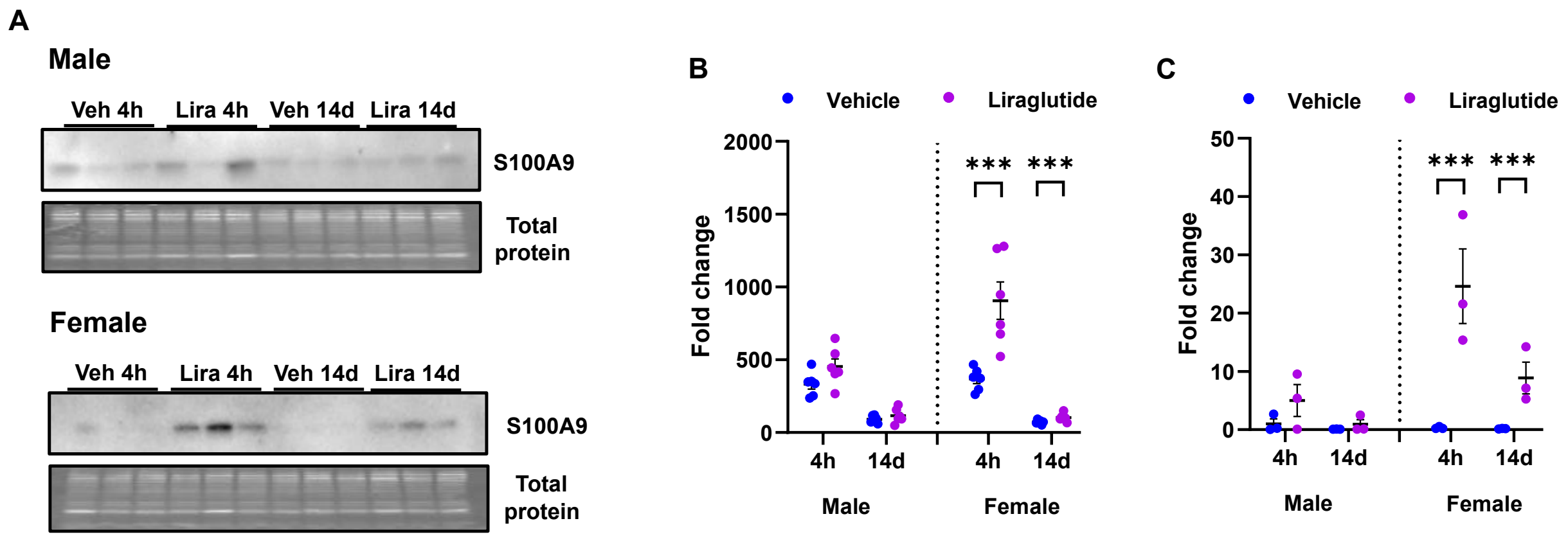

Figure S4. Western blots of S100A9 immunoreactive bands in neurointermediate lobe samples of acute (4 hours) and chronic (14 days) male and female control and liraglutide-treated rats **(A)**. **B**, proteomic data for S100A9 expression in neurointermediate lobe samples of acute and chronic male and female control and liraglutide-treated rats. **C**, western blot analysis of S100A9 expression in neurointermediate lobe samples of acute and chronic male and female control and liraglutide-treated rats. The total protein stain No-stain was used for the normalisation of western blotting data. Statistical analyses were performed by independent-sample unpaired t tests. Values are means ± SEM of n = 3 - 6 biological replicates per group. ***$p \leq 0.001$.

Figure 7C

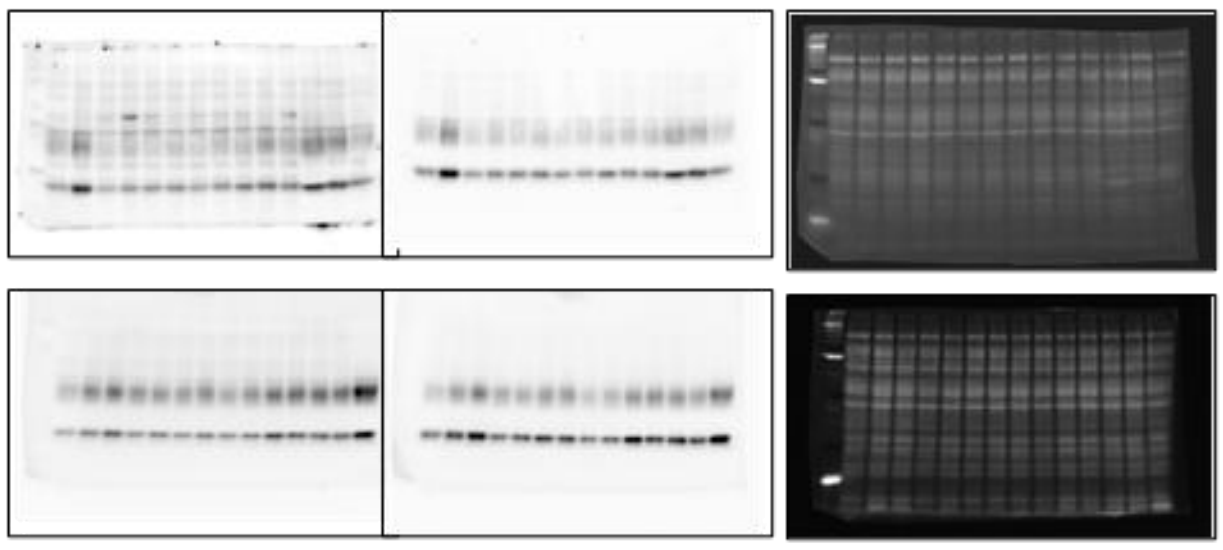

Figure 7D

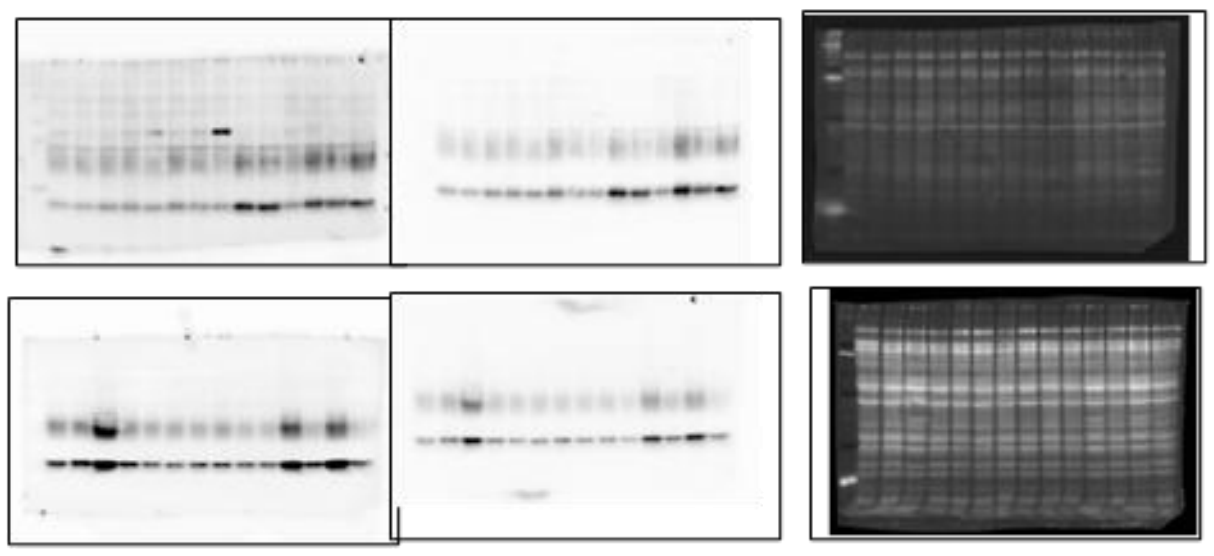

Figure 7E

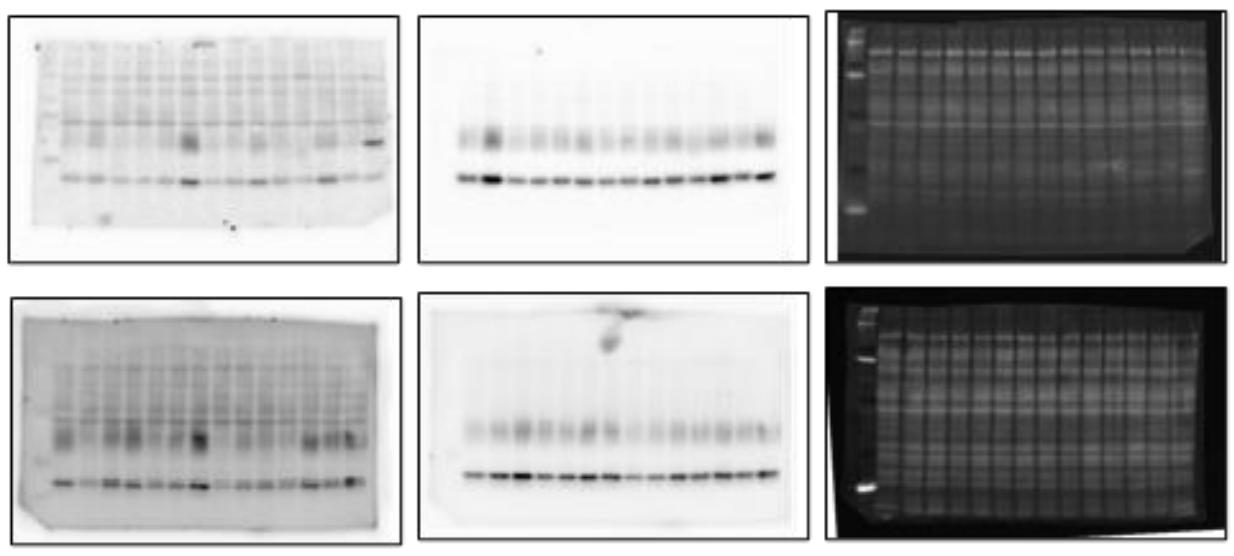

Figure 7F

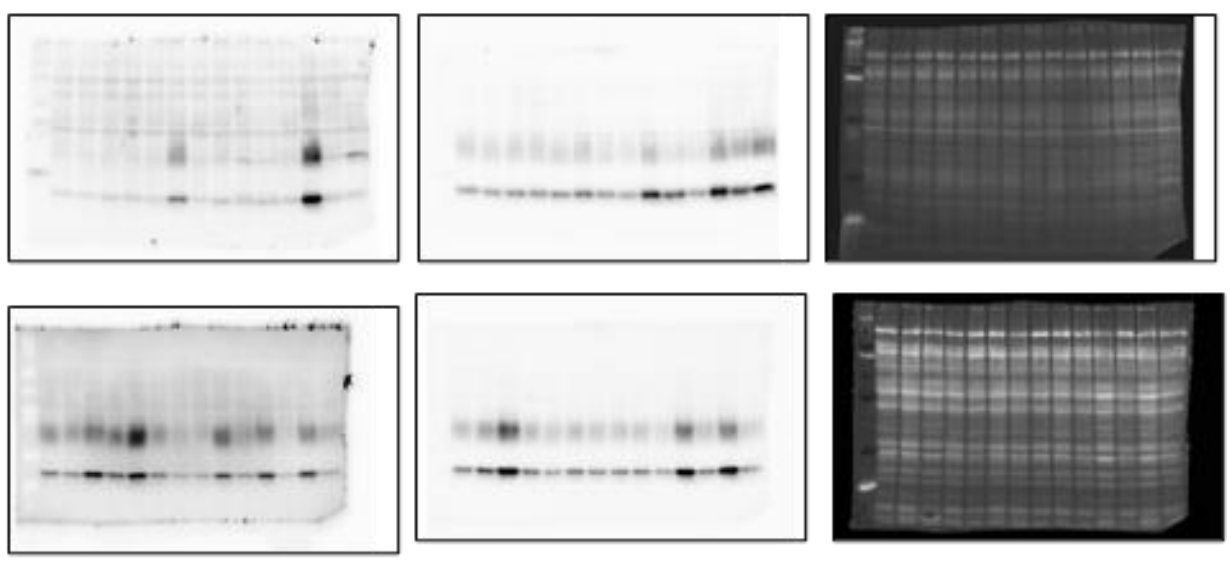

Supplemental Figure 4.

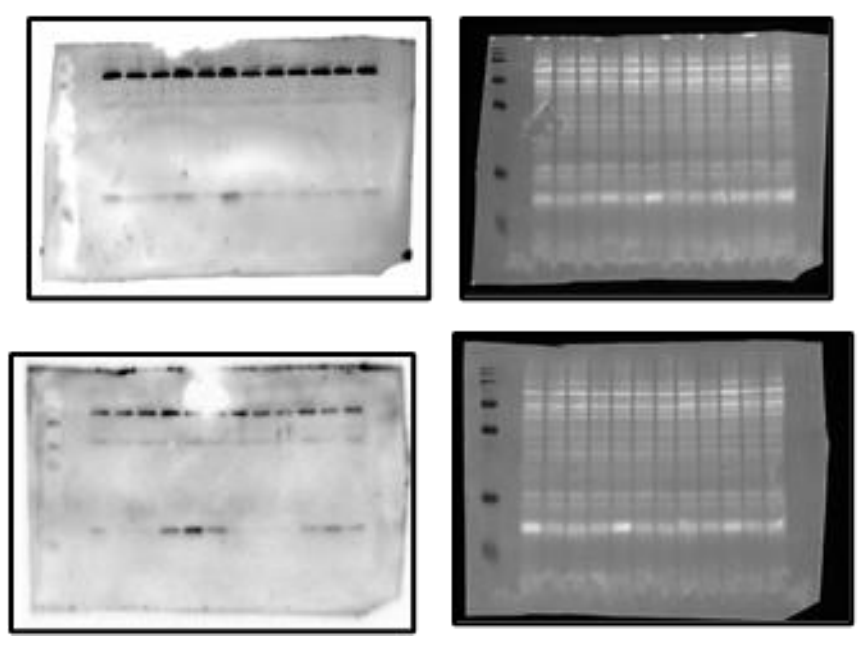

Figure S5. Western blot full membrane images.
